# The interaction of genetic sex and prenatal alcohol exposure on health across the lifespan

**DOI:** 10.1016/j.yfrne.2023.101103

**Published:** 2023-10-04

**Authors:** Shameena Bake, Siara K. Rouzer, Shruti Mavuri, Rajesh C. Miranda, Amanda H. Mahnke

**Affiliations:** Department of Neuroscience and Experimental Therapeutics, Texas A&M University School of Medicine, Medical Research and Education Building I, 8447 Riverside Parkway, Bryan, TX 77807-3620, United States

**Keywords:** Prenatal alcohol exposure, Sex differences, Adolescence, Adulthood, Cardiovascular disease, Mental health, Alcohol use disorder

## Abstract

Prenatal alcohol exposure (PAE) can reprogram the development of cells and tissues, resulting in a spectrum of physical and neurobehavioral teratology. PAE immediately impacts fetal growth, but its effects carry forward post-parturition, into adolescence and adulthood, and can result in a cluster of disabilities, collectively termed Fetal Alcohol Spectrum Disorders. Emerging preclinical and clinical research investigating neurological and behavioral outcomes in exposed offspring point to genetic sex as an important modifier of the effects of PAE. In this review, we discuss the literature on sex differences following PAE, with studies spanning the fetal period through adulthood, and highlight gaps in research where sex differences are likely, but currently under-investigated. Understanding how sex and PAE interact to affect offspring health outcomes across the lifespan is critical for identifying the full complement of PAE-associated secondary conditions, and for refining targeted interventions to improve the quality of life for individuals with PAE.

## 1. Introduction

Prenatal alcohol (ethanol) exposure (PAE) is very common, as demonstrated by studies using objective biochemical markers for alcohol exposure, such as blood phosphatidylethanol (PEtH). For instance, a study assessing the prevalence of PAE in women seeking routine prenatal care in the Netherlands documented an exposure rate of 5.3% during the 1st trimester of pregnancy (Breunis et al., 2021). In the US and Canada, statewide studies in the general population have assessed PEtH levels in newborn infant blood collected for routine genetic screening. These studies uncovered PEtH-positivity in 8–15% of infants assessed, indicative of prenatal exposure during the last month of pregnancy (Bakhireva et al., 2017; DiBattista et al., 2022; Umer et al., 2020). Prevalence estimates of pregnancy-related alcohol exposure are substantially higher in populations with high societal rates of alcohol use (Baldwin et al., 2020). Unsurprisingly, prevalence estimates for fetal alcohol spectrum disorders (FASDs) are similarly high. A recent prospective case-ascertainment study in four US school systems estimated a conservative FASD prevalence of 1.1–5% of school-aged children and a weighted estimate of 3.1–9.8% (May et al., 2018). The global prevalence of FASDs is estimated at ~ 2.3%, with regional highs of 11.3% of the general population (Roozen et al., 2016).

FASDs include a spectrum of brain-based disabilities, including deficits in memory, cognition, sensorimotor integration, and executive functioning. Sex and gender differences in PAE outcomes have been previously well reviewed in book chapters discussing data from animal models (Otero et al., 2012) and human studies (Streissguth et al., 2012). Since the publication of these reviews, additional research has further assessed the explicit contribution of biological sex to the presentation of FASD symptoms. For instance, in a 2017 study (May et al., 2017), male children with FASDs had diminished viability at birth and decreased overall survival compared to females. While the authors noted that there was a general similarity in FASD symptom presentation between females and surviving males, female children exhibited significantly more severe dysmorphia, i.e. altered facial structure and neurobehavioral outcomes. While these data may have been influenced by a ‘survivor bias’, they do suggest that sex differences drive vulnerability to the physical and neurobehavioral consequences of PAE. Given that components of facial dysmorphology have been attributed to alcohol exposure around gastrulation (early 1st trimester-equivalent) in both rodent and non-human primate models (Astley et al., 1999; Sulik, 2005), these findings strongly suggest that sex differences in vulnerability to PAE’s impacts can arise early within the *in utero* period.

Seminal work by David Barker and colleagues found an association between infant health/viability and health in adulthood (Barker and Osmond, 1986). In the UK, regional adult mortality rates for cardiovascular disease (CVD), some cancer types, and chronic bronchitis were highly correlated with rates of infant mortality 45 years prior, a proxy marker for overall infant health, indicating that health during fetal life can determine health across the lifespan. This work led to the “Barker Hypothesis”, which is now known as the “developmental origins of health and disease” (DOHaD), a hypothesis that is supported by a substantial published literature [reviewed in Hoffman et al., 2017]. CVD is one of the most researched DOHaD-associated diseases. However, there are known age-by-sex interactions that impact the morbidity and mortality of CVD [reviewed in Mosca et al., 2011].

**Fig. 1.**
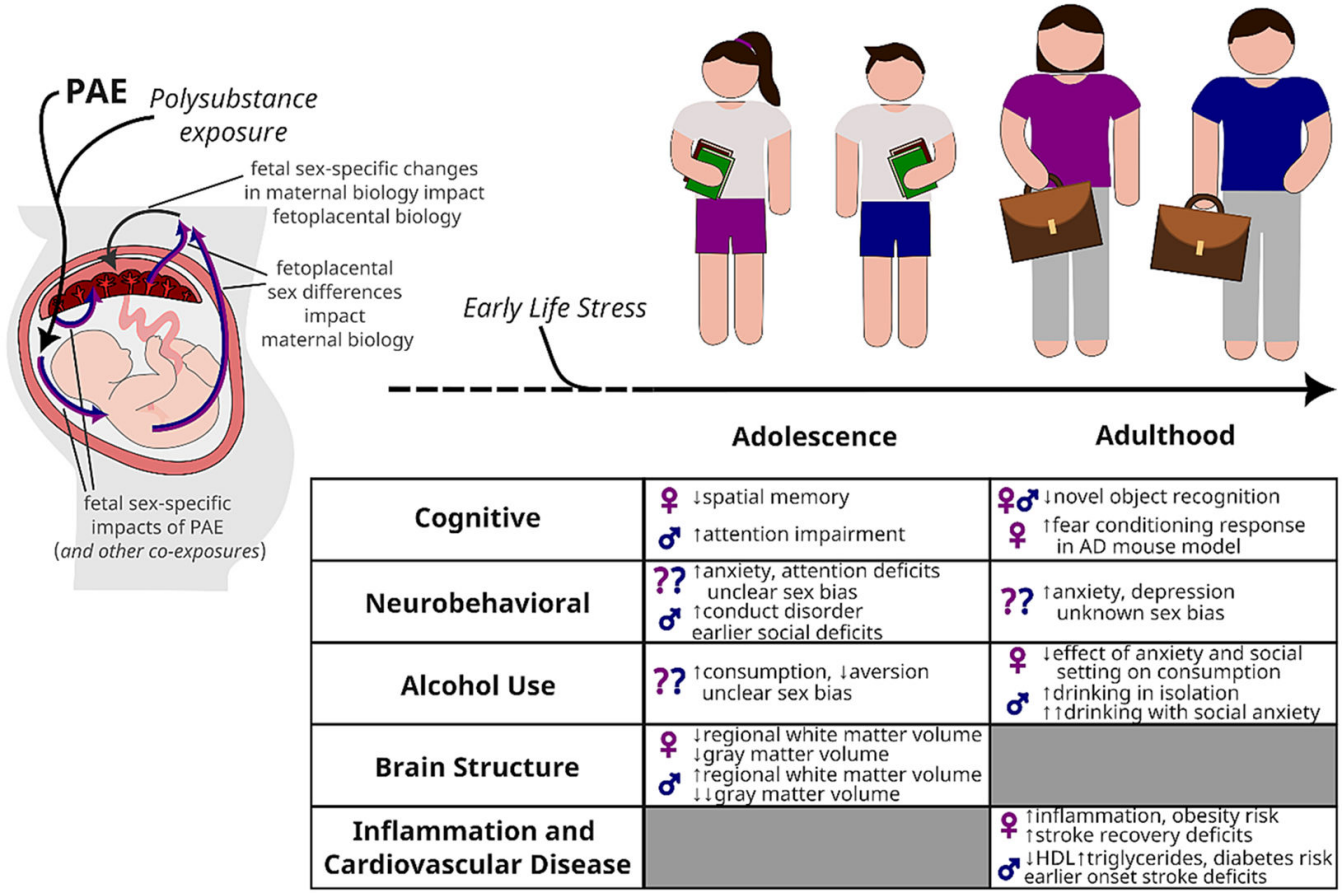
Developmental origins of sex differences in PAE’s effects during adolescence and adulthood. Sex differences in PAE’s effects originate during the *in utero* period and are likely futher shaped by polysubstance exposure and early life stress. PAE results in long-term changes to cognition and health, with some effects presenting with age- and sex-bias. Shown are the currently known sex-differences in PAE outcomes in adolescence and adulthood. Italics denote modifiers of PAE’s impacts that need additional research.

Similarly, as we will describe below, sex differences in the effects of PAE can arise during the *in utero* period and appear throughout the lifespan of offspring (Fig. 1). Studies of PAE that do not investigate sexually dimorphic symptoms may not be positioned to uncover exposure-by-sex interactions due to unequal representation between males and females (O’Connor et al., 2002) or insufficient power due to a limited sample size (Fryer et al., 2007; Mariasine et al., 2014; Fryer et al., 2007). Even studies with suitable sample size and sex representation do not always report offspring outcomes separately for males and females (Olson et al., 1997; Streissguth, 2007), limiting our ability to determine whether offspring are predisposed to sex-specific PAE-induced outcomes. However, more recent and considerable efforts have been made to stratify by sex, revealing distinct outcomes in male and female offspring across species, and notably, the lifespan. Here, we not only discuss known sex differences in PAE-related outcomes, but also reference research where both sexes were represented without =statistical assessment of exposure-associated outcomes. Existing literature on various neurodevelopmental disorders can further emphasize the role of sex as an important, yet underexplored, factor influencing the outcomes of alcohol exposure during prenatal development.

## 2. Developmental origins of lifelong impacts of PAE

### 2.1. Fetal sex-specific alterations to maternal biology

Fetal sex impacts maternal biology in uncomplicated pregnancies as well as pregnancies with complications. Male-bearing pregnant women have higher levels of proinflammatory cytokines in circulation (Goldstein et al., 2021; Enninga et al., 2015), whereas female-bearing pregnancies have enhanced cytokine release following inflammatory challenge, such as exposure to lipopolysaccharide (Mitchell et al., 2017). Female fetus-specific enhanced stimulated cytokine release also occurs in pregnancies with asthma as a co-occurring complication. Female-bearing pregnant women with asthma experience a higher risk for exacerbation of asthma symptoms, with higher rates of hospitalization for asthma (Bakhireva et al., 2008), increased inhaled glucocorticoid use across pregnancy (Murphy et al., 2003), and elevated inflammatory tone, indicated by higher numbers of circulating monocytes (Murphy et al., 2003), compared to male-bearing pregnant asthmatic women.

Sex-specific modulation of maternal immune profiles is associated with long-term health outcomes in offspring. In an assessment of adults from the New England Family Study, who were followed from the *in utero* period into adulthood (average age of assessment in adulthood: 45.9 years), alterations to brain regional activity and connectivity in adulthood were associated with altered maternal proinflammatory cytokine levels at the start of the third trimester (Goldstein et al., 2021). As adults, subjects underwent functional magnetic resonance imaging (fMRI), and regional brain activation during presentation of negative-valence images was compared to neutral-valence images. Overall, expression of proinflammatory tumor necrosis factor (TNF)-α in maternal sera was associated with a larger change in hypothalamic activation during negative image presentation in adulthood. The ratio of maternal sera proinflammatory TNF-α to anti-inflammatory interleukin (IL)-10 was associated with elevated hippocampal activation in female adult offspring and decreased hippocampal elevation in male adult offspring. These data suggest that the maternal cytokine milieu is reflective of, and potentially a contributor to, lifelong sex-specific differences in brain function and connectivity.

Likewise, PAE alters the maternal milieu of plasma cytokines (Bodnar et al., 2018) and plasma/serum microRNAs (miRNAs) (Balaraman et al., 2016; Gardiner et al., 2016), small non-coding RNAs that can also act as pro- or anti-inflammatory endocrine molecules. Altered maternal cytokines and miRNAs predicted child outcomes following PAE; specifically, growth deficits and neurodevelopmental delay at 6 and 12 months of age (Bodnar et al., 2018; Balaraman et al., 2016). Networks of altered maternal cytokines were associated with alcohol exposure and offspring PAE-dependent and independent neurodevelopmental delay (Bodnar et al., 2018). Intriguingly, during the second trimester, a network of cytokines indicative of PAE included the maternal expression of TNF-α and IL-10. This, in light of the work by Goldstein and colleagues (Goldstein et al., 2021), may be indicative of PAE-altered sex differences in offspring hippocampal brain activity in adulthood. Altered maternal plasma miRNAs predicted infant outcomes and were associated with decreased growth, collectively explaining 13–32% of the variance in infant growth measurements (Tseng et al., 2019). Preclinically, outcome-predictive miRNAs directly inhibited fetal and placental growth in pregnant mouse dams in the absence of alcohol exposure (Tseng et al., 2019).

Given that fetal sex has been shown to shape signaling molecules in maternal circulation, these alcohol-altered maternal cytokine and miRNA profiles are likely shaped by fetal sex. Indeed, in a reanalysis of the maternal miRNA data, fetal sex-specific alterations to the maternal miRNA profile were identified (Salem et al., 2020). Using an iterative statistical sampling method, bootstrap resampling with replacement, maternal miRNA profiles could be disaggregated by fetal sex to examine fetal sex contributions to maternal miRNA expression. The majority of alcohol-associated maternal miRNAs were not altered by fetal sex; however, some miRNAs were more likely to be significantly altered by alcohol when the sample was disaggregated by fetal sex. The degree of correlated miRNA expression, which could be indicative of intracellular mechanisms of co-expression or cross tissue co-secretion, was also assessed. In the second and third trimesters for both fetal sexes, pregnancies with heavy alcohol exposure and infants who exhibited PAE symptoms had more miRNAs with significantly correlated expression than a) pregnancies with heavy alcohol exposure, where the infants did not exhibit PAE symptoms, and b) pregnancies with little to no alcohol exposure. However, in the second trimester, the increased correlation was driven by female-bearing pregnancies. These data indicate an earlier vulnerability for altered maternal miRNA milieu following alcohol exposure in pregnancies with female fetuses.

### 2.2. Fetal sex-specific placental functions

While the identified alcohol-sensitive miRNAs and cytokines may derive from maternal tissues, the placenta is also a major endocrine organ [reviewed in Costa, 2016]. Endocrine factors can not only be released into maternal circulation, but also into the fetal compartment, impacting fetal development. For example, in pregnancies with co-occurring asthma, female fetuses, but not male fetuses, had significantly reduced birth weights (Murphy et al., 2003). This altered birth-weight was negatively associated with umbilical vein plasma cortisol levels and occurred alongside elevated placental cytokines (Scott et al., 2009). Cortisol levels were also predictive of increased expression of the pro-inflammatory cytokine, TNF-α, in the female placenta (Scott et al., 2009), suggesting that altered cytokines in the placenta are part of the altered physiology that contributes to female growth restriction in asthmatic pregnancies.

Sex differences identified in the placenta include sex-specific gene expression and hormone and immune signaling [reviewed in Braun et al., 2022]. For example, among the ~ 14,000 genes expressed in first-trimester chorionic villi, 0.4% were found to have significantly different expression between male and female placentae (Gonzalez et al., 2018). Of the identified 58 genes with sex-specific expression patterns, 31% were encoded in autosomes, indicating that sex differences in gene expression are not restricted to sex chromosomes. The magnitude of sex differences in placenta likely vary across gestation. For example, one study in mice uncovered reduced labyrinthine volume in female placentae compared to male placentae on gestational day (GD) 15, but not earlier, at GD 13, or later, at GD 20 (Kalisch-Smith et al., 2017), highlighting potential critical periods for sex differences in placental development.

PAE affects both placental development and function. In a *meta*-analysis of clinical studies, PAE decreased placental weight by 51 g (approximately 10% of placental weight) and increased risk for placental abruption [odds ratio 1.48, Steane et al., 2021]. Altered placental gene and protein expression have been found in both clinical and preclinical studies following PAE (see Table 1 for genes and proteins identified in multiple studies). These studies show that some, but not all, of the placental genes and proteins impacted by PAE are likely altered in a sex-specific manner. For example, for glucose transporter GTR3 mRNA, assessments in sex-segregated samples indicate that PAE induces not only sex-specific alterations, but also age- and region-specific alterations. Additional studies powered to examine sex differences have identified altered expression of genes with sex-biased expression, such as machinery for the female phenomenon of X chromosome inactivation, including the E3 ubiquitin-protein ligase *RLIM* and the long non-coding RNA *Xist*. Contrastingly, programmed cell death 10 (PCD10) and placental growth factor (PLGF) both exhibited decreased placental expression following PAE across multiple studies and model systems. Although assessments of PCD10 and PLGF have not been segregated by sex, the robust effect across studies indicates that these differences are likely sex-independent. However, sex-segregated analysis of these genes could reveal a difference in the magnitude of gene expression change by sex. Furthermore, for genes with conflicting results across studies, sex-segregated analyses may identify a sex-confounded gap in literature, and provide a clearer understanding of PAE’s impacts on placental gene expression.

Sex differences in epigenetic factors are likely upstream of some sex-specific alterations in gene expression. Pinson and colleagues (Pinson et al., 2022) found that following a single, binge-like ethanol exposure on GD 10.5, DNA demethylation enzyme *Tet3* was significantly upregulated in male, but not female, placentae, which could lead to altered epigenetic regulation, and therefore altered expression of additional genes. In a clinical cohort, sex-specific alterations to the epigenetic landscape have also been identified in the placenta following PAE (Loke et al., 2018). Samples from term placenta were assessed for global methylation and no differences were found between placentae with PAE compared to those without PAE. However, after stratification by sex and adjustment for sex-specific confounders (i.e. variables that were associated with PAE and methylation, including maternal age and quality of life in male offspring), male-bearing, but not female-bearing, pregnancy-derived placenta had higher global methylation when PAE occurred throughout pregnancy compared to non-exposed placenta. These data indicate that PAE induces sex-specific gene and protein expression in placentae, in part, through altered epigenetic regulation.

These sex-specific changes in placental gene expression may reflect adaptive or maladaptive responses to PAE. Furthermore, they may drive some of PAE’s functional consequences for pre- and post-parturition health outcomes. In a periconceptional exposure rat model (Kalisch-Smith et al., 2019), altered gene expression (e.g. see Table 1) occurred alongside reduced placental volume, maternal blood space, and trophoblast DNA content, indicative of reduced cell number, at the intersection of the junctional zone and the decidua in female placentae at GD 15. These findings occurred during a window of diminished female fetal viability, suggesting that impaired placentation in females may play a role. Additionally, as discussed further below (see Section 5), the stress axis for individuals with PAE is not only usually subjected to more stress, but may be reprogrammed during fetal life to aberrantly respond to these stressors. PAE throughout rat gestation (GD 1 – GD 21) decreased placental efficiency, or the fetal:placental weight ratio, and altered maternal/fetal endocrine signaling through elevation of maternal cortisol, maternal progesterone:estradiol ratio, and placental protein expression of 11β-hydroxysteroid dehydrogenase type 2 (11β-HSD2) (Lan et al., 2017). 11β-HSD2 is an enzyme that converts the stress hormone corticosterone into an inactive form. Increased expression of placental 11β-HSD2 at the end of rodent *in utero* gestation has been repeatedly observed (see Table 1) and indicates that PAE limits the bioavailability of maternal cortisol within the fetoplacental compartment. While the increase in 11β-HSD2 was sex-independent in this study, i.e. 11β-HSD2 was significantly elevated in male and female placentae compared to sex-matched controls and a prenatally stressed group (GD 11- GD 20), there were sex-specific changes in fetal cortisol receptor expression. For pregnancies with male fetuses, but not female fetuses, maternal alcohol consumption was negatively associated with placental protein expression of the mineralocorticoid receptor, one of the corticosteroid receptors, indicating sex-biased dysregulation of placental stress hormone signaling following PAE.

It should be noted that prenatal exposure to synthetic glucocorticoids mimics some of the effects seen following PAE [impact of prenatal synthetic glucocorticoid exposure reviewed in Goldstein et al., 2016], suggesting that actions of PAE to reprogram the developing hypothalamic–pituitary–adrenal (HPA) axis may be due to a maternal stress response and not direct pharmacological action of PAE. Interestingly, the aforementioned work by Dr. Joanne Weinberg’s group (Lan et al., 2017) found an elevation of placental 11β-HSD2 that was unique to PAE and not seen in prenatal stress. Similarly, work in the laboratory of Dr. Karen Moritz has examined placental expression of *Hsd11b2*, the mRNA for 11β-HSD2, following corticosterone administration (GD 12.5) and following periconceptional alcohol exposure (from 4 days prior to conception to GD4). In these murine models, administration of corticosterone increased *Hsd11b2* expression at GD 14.5 in both sexes (Cuffe et al., 2012). By GD 17.5, this effect was no longer seen in female placenta and male placenta exhibit decreased expression. These effects mimic, but imperfectly phenocopy, periconceptional alcohol exposure, which results in a sex-independent increase in placental *Hsd11b2* later in gestation (GD 20) (Gårdebjer et al., 2014). Similarly, corticosterone administration enlarged male placenta (Cuffe et al., 2012), while periconceptional alcohol exposure decreased female placenta volume (Kalisch-Smith et al., 2019). While the timing of glucocorticoid exposure and PAE can alter the profile of outcomes, making these comparisons imperfect, these data suggest that maternal stress, as modeled by corticosterone administration, underlies some, but not all, of PAE’s effects.

Collectively, these data indicate that PAE induces sex-specific programming of the fetal HPA axis. This has sex-specific consequences for stress responsivity across the lifespan [reviewed in Weinberg et al., 2008] and is partially attributable to a PAE-induced maternal stress response, but also exacerbated by PAE’s direct actions on pathways complementary to the stress response. Additional research is required interrogate PAE’s actions as a stressor, verses direct pharmacological actions, and the contribution of each to sex-specific outcomes and reprogramming of the stress response axis.

### 2.3. Fetal sex-specific somatic cell reprogramming

PAE does not only reprogram cells within the placenta, but within the fetus as well. PAE has been shown to reprogram stem cells across the body plan [reviewed in Mahnke et al., 2018], which may contribute to a high number of co-morbid conditions for individuals with PAE (Himmelreich et al., 2020; Popova et al., 2016). This cellular reprogramming can occur in a sex-biased manner during fetal development. For example, in a PAE mouse model, a single ethanol exposure at GD 12.5 altered the developing neocortical transcriptome at GD 14.5 (Salem et al., 2021). For female cells, long non-coding RNAs (lncRNAs) that control X chromosome inactivation, *Xist* and *Tsix*, were found to be downregulated and upregulated, respectively. Given the known role in X chromosome inactivation of these lncRNAs, with *Xist* triggering inactivation of one X chromosome and *Tsix* preventing inactivation of the other chromosome, it is not surprising that these PAE-induced changes upregulated the expression of genes across the X chromosome. In addition, some gene expression on autosomes was also correlated with changes in expression to *Xist*; bioinformatics analysis suggested that *Xist* is a hub gene for PAE-induced changes in the transcriptome in a subpopulation of neural stem cells. These data suggest that a mechanism exists by which *Xist* acts in trans to control gene expression on other chromosomes, either directly or indirectly. This female-specific mechanism of PAE effects may not be limited to the developing cortex, as periconceptional alcohol exposure also decreased *Xist* expression in female rat placentae at GD 13 (Kalisch-Smith et al., 2019)(Table 1). Additional sex-dependent PAE reprogramming occurs in extracellular vesicles released by neural stem cells, at both the transcript and protein levels. Proteomic assessment in GD 12.5-derived fetal mouse cortical neural stem cells, cultured *ex vivo*, and their released extracellular vesicles (EVs) found that a network of EV-associated proteins was altered by the fetal sex of the cell-of-origin (Chung et al., 2022), suggesting that sex differences exist in EV-mediated endocrine transfer of proteins and other information between cells. In response to ethanol, female neural stem cells exhibited increased rates of oxidative metabolism, while male neural stem cells exhibited decreased rates of glycolysis, suggesting that additional basic cellular biological mechanisms are affected by sex. Surprisingly, at the transcript level, sex was a major driver of transcriptomic differences in neural stem cells and their EVs (Chung et al., 2023). Principal component analysis found that location of transcript, *i.e.* whether in cells or EVs, explained 53% of sample transcriptomic variance and that the next highest contributor to variance was fetal sex (9.3% of variance).

*In utero* sex-specific programming sets up a trajectory of postnatal sex differences. For example, miRNAs were identified in infant plasma at 2 weeks and 6.5 months of age that had altered expression following PAE (Mahnke et al., 2021). Using the same statistical method that identified fetal sex-dependent alterations to maternal miRNAs (Salem et al., 2020), likely sex-specific miRNAs were identified. There was a pronounced increase in the number of likely sex-specific miRNAs from 2 weeks to 6.5 months of age, suggesting that sex differences become more apparent over development. Sex differences in the impact of PAE in early childhood/development have been noted by others (Otero et al., 2012; Streissguth et al., 2012; May et al., 2017). We highlight below the longer-term sex-dependent effects of PAE that extend into adolescence and adulthood.

## 3. Sex-specific outcomes following PAE across adolescence and adulthood

The lifelong consequences of PAE span many physical and psychological domains, including cognitive and neurobehavioral deficits, altered alcohol-seeking behavior, and increased risk for CVD and stroke. Here, we focus on investigations of offspring within early life/adolescence and adulthood. Adolescence denotes an epoch of life beginning with the onset of puberty (~11–12 years old) and ending with the transition into young adulthood (~24 years old), and serves as a prominent period of biological, psychological and emotional development in humans (National Academies of Sciences, Engineering, and Medicine et al., 2019). Life experiences during this early period can change the trajectory of health outcomes observed in adults. Furthermore, sexual dimorphism resulting from developmentally maturing neuroanatomy, hormonal contributions and/or life experiences during adolescence, can interact with PAE to impose unique outcomes on male and female offspring. Therefore, adolescence and adulthood represent two windows where there can be: 1) disparate impacts of PAE due to differing vulnerabilities across age, or 2) a trajectory of PAE effects that appear in adolescence and further develop into adulthood.

### 3.1. Cognitive outcomes

Impaired cognitive performance occurs across the lifespan for individuals with FASDs. A records-review study from Canadian FASD clinics examined co-occurring neurodevelopmental impairment for individuals with FASDs across a wide span of ages: 53% were school age or younger, 23% were adolescents, and 24% were adults (Flannigan et al., 2023). Males with FASDs had higher rates of impairments than females with FASDs for motor skills, memory, attention, and adaptive function, after controlling for age. Moreover, life experiences for individuals with PAE during adolescence can shape adult cognitive function. PAE and chronic mild stress during adolescence contributed to high numbers of errors in radial arm maze exploration in adult (~4-month-old) Sprague-Dawley rat females, but not males (Comeau et al., 2015). The adolescent and adult epochs also have unique vulnerabilities that can impact cognitive performance, such as the adolescent ability to integrate into/perform in school and later adulthood age-related cognitive decline, for which PAE has sexually dimorphic effects.

#### 3.1.1. Adolescence

Among the general population, adolescent cognitive function and behaviors are sexually dimorphic. Overall, adolescent females typically demonstrate greater academic prowess than age-matched males (Peper et al., 2020). Compared to age-matched (10–16 years) boys, girls have received higher ratings for social and emotional skills, indicating more close, compassionate, and supportive relationships (Head et al., 2014). This finding was replicated by a second study that specified female-favoring social acuity was most prominent after pubertal onset (Gur and Gur, 2016). Assessments of older adolescents (17–20 years) using the Montreal Cognition Assessment Test found that female subjects scored significantly higher than male subjects in visuospatial and memory skills, while male subjects outperformed female subjects in mathematical abilities (Mittal et al., 2012). Sex differences in executive function, in which girls excel in attention while boys excel in working memory speed, begin to emerge after age 11 (Gur and Gur, 2016). Notably, these sex differences during adolescence do not always continue into or predict cognitive ability in adulthood. In an assessment of cognitive bias (generalizing a new environment to a negative rather than positive context), middle-aged male rats displayed more negative bias than females (Hodges et al., 2022). This sex difference in bias was not observed in adolescence or young adulthood. Furthermore, behavior in this task was associated with sex-specific neural activity that changed across age. For females, cognitive bias was associated with: decreased c-Fos expression in the dorsal hippocampal CA3 in adolescence, increased c-Fos expression in the infralimbic cortex in young adulthood, and decreased c-Fos in the ventral hippocampal dentate gyrus and CA1 in middle-age. Males displayed no significant relationships between cognitive bias and c-Fos expression at any age.

In general, PAE is associated with declines in adolescent cognitive performance, as children and their parents have reported a multitude of learning problems associated with PAE, including poor academic progress and greater enrollment in supplemental learning programs (Olson et al., 1997). Furthermore, some children with PAE can have significantly lower IQs than aged-matched children without PAE (Streissguth, 2007; Howell et al., 2005). Despite overarching sex differences in cognitive performance in the general population, investigations of PAE-associated cognitive performance that include sex as a biological variable are notably limited (Table 2). In a recent analysis of clinical records from FASD clinics in Canada (Flannigan et al., 2023), which examined cognitive impairment across the lifespan for individuals with FASDs, adolescent males with FASDs exhibited greater attention impairments and school problems (involving a teaching assistant or suspension/expulsion) than adolescent females. Although these academic issues were not further interrogated in this study, the investigators acknowledge that sex-specific challenges in adaptive and executive function, and subsequently scholarly performance, may underly this male-favoring phenotype.

Among preclinical investigations of PAE that analyze sex differences in young offspring, several have emphasized the susceptibility of female subjects to spatial memory deficits. In late adolescent rats (postnatal day [PND] 40–48), chronic ethanol administration resulted in greater sensitivity to alcohol impairment in efficient maze exploration, requiring more time to navigate the radial arm maze and extinguish a formerly correct maze entry, in female rats compared to male rats (Maier and Pohorecky, 1986). These data modeling adolescent alcohol exposure mimic what has been seen in adolescence following PAE. A study in juvenile Long-Evans rats (PND 22) found that PAE females perform worse in the Morris water maze task, i.e. they took longer to reach the platform, than PAE males. However, PAE males scored significantly lower in the strategy to find the platform (Blanchard et al., 1987), suggesting they are not immune to all PAE-associated deficits during this developmental period. This idea is further supported by one study of spatial memory, in which both male and female PAE offspring (PND 36–39) exhibited impaired performance on the Morris water maze compared to control offspring, but engaged in different, sex-specific behaviors that prolonged maze navigation (An and Zhang, 2013). These distinct swimming behaviors corresponded with opposing effects of PAE on hippocampal long-term potentiation, which was significantly reduced in males but facilitated in females (An and Zhang, 2013). An additional study has reported similar potentiation of LTP in adolescent (PND 30–35) female rats and reduction of LTP in males compared to their respective ad libitum, but not pair-fed, controls (Titterness and Christie, 2012). Together, these data may highlight a synaptic imbalance that imposes unique sex-specific deficits in adolescent offspring.

To better understand these adolescent sex difference in cognitive function following PAE, more work is needed to assess co-occurring conditions that impose sex-biased barriers to learning in adolescents with PAE. Investigation of sex by PAE interactions are futher needed in areas that include hearing loss and auditory processing deficits (Simões et al., 2023), challenges with reading and communication both oral and through written t (Lindinger et al., 2022; Kippin et al., 2021), and changes in brain lateralization and neural function (Yu et al., 2022) (for known sex-differences in PAE-induced alterations adolescent brain structure, see section 4.1). Other environmental factors, including geographic location and nutritional status, are further believed to influence gender differences in school-aged test performance (Thuc Duc and Behrman, 2021), and have not been well-assessed as a moderating factor for later life outcomes following PAE. Additionally, known sexually dimorphic outcomes following PAE likely contribute to sex differences in adolescent cognitive function. PAE-associated changes in endocrine function, such as elevated testosterone in adolescent males and females with PAE, may impact cognitive performance (Carter et al., 2014), as pubertal testosterone levels have been negatively associated with spatial reasoning performance in young adult males (Vuoksimaa et al., 2012). Distinct to each sex, individuals with FASDs are more susceptible to experiencing certain adolescent life events, which may contribute to sexually dimorphic outcomes. This includes legal problems and trauma, which are more likely to occur in females with FASDs than males, while males with FASDs face greater difficulties with school and incarceration than females (Flannigan et al., 2023). Female children and adolescents with a history of PAE are also more likely to experience sexual abuse than male counterparts (Flannigan et al., 2021).

#### 3.1.2. Adulthood

Investigations of the impact of PAE on cognitive performance later in life have repeatedly revealed, through clinical and preclinical studies, abnormal declines in learning and memory performance during adulthood. In humans, a pair of studies have identified early adulthood cognitive deficits for individuals with PAE/FASD. In young adults (mean age: ~23 years), both verbal and non-verbal memory are impaired by PAE, as determined by the Verbal and Nonverbal Selective Reminding Memory Tests (Coles et al., 2011). Separately, adults with FAS (n = 20/group, mean age: 30 years), display reduced cognitive, executive, and social function compared to non-exposed controls, as determined by their performance on Raven’s Colored Progressive Matrices, a digit span task, and Wisconsin card sorting test (Rangmar et al., 2015). Unfortunately, neither of these studies stratified PAE outcomes by sex. In pre-clinical models, PAE has similarly produced deficits in adult spatial processing, including sensing and consolidating information to navigate spaces, and in the recall of familiar environments and cues, across a multitude of cognitive tasks (as reviewed extensively in (Harvey et al., 2019).

The bulk of this research does not expressly investigate or report the outcomes of cognitive performance by chromosomal sex, and those studies which have reported PAE effects in both males and females (Table 2) have reported inconsistent and variable outcomes. A few preclinical studies have suggested that adult males are uniquely vulnerable to PAE-induced deficits in spatial learning and memory. In a model of early postnatal exposure to alcohol (roughly equivalent to the third trimester in humans), only adult (PND 77–98) male mice with PAE displayed memory impairments navigating the Morris water maze (Ieraci and Herrera, 2020). Adult (PND 105–112) males with PAE were also the only group to experience less freezing time in a context fear conditioning paradigm than control counterparts (Ieraci and Herrera,2020), indicating an inability to recall prior exposure to a foot shock. Separately, adult (PND 75–100) male rats with PAE made significantly more errors when navigating a T-maze compared to controls, a deficit that was not observed in PAE females (Zimmerberg et al., 1991). However, the opposite sex-bias has also been reported for young adult mice. Moderate PAE reduced performance in a hippocampal-dependent visual-spatial discrimination task in young adult (~PND 56–63) female, but not male, mice compared to sex-matched controls (Kenton et al., 2020). Similarly, young adult (PND 58–60) female PAE rats exposed to alcohol during the final week of gestation took significantly longer to learn a radial arm maze task than sex-matched offspring exposed during either earlier week of gestation (Neese et al., 2004). Additionally, female offspring exposed to alcohol throughout gestation made significantly more mistakes in completing the maze than female offspring without PAE. Male offspring notably showed no spatial working memory deficits for any of these exposure windows. These data suggest that sex differences in cognition in young adulthood are more similar to adolescents, with PAE females showing more deficits, than later adulthood, where PAE males show more deficits. However, some studies have reported consistent deficits between males and females with PAE. In one study, adult (~PND 135) male and female adult rats with PAE displayed similarly poor recognition memory in the novel object recognition task (Bake et al., 2021). Another study uncovered comparable reductions in spatial recognition memory between male and female adult (PND 90) offspring with PAE (Subbanna and Basavarajappa, 2022). Collectively, these data suggest that there are windows of vulnerability for exposure to induce sex-differences in adult cognition and that assessed differences in PAE’s effects on cognition vary across adulthood. Therefore, future research incorporating sex in statistical analyses will be necessary to refine our understanding of which cognitive tasks are sex-specific, or sex-independent and at what ages.

Importantly, neurological processes underlying cognitive ability have been demonstrated to differ between male and female offspring. Physiological assessments of long-term potentiation (LTP) - a strengthening of synaptic connections associated with learning - determined that PAE reduced LTP in the hippocampus of adult (PND 55–70) male, but not female, offspring (Sickmann et al., 2014). Notably, PAE produced similar effects between ovariectomized females and ovary-possessing females, which suggests that estrogen was not the driving force behind these sex-specific findings. PAE females may have been protected by a compensatory increase in glutamine synthetase levels that was not observed in male counterparts. However, given their aforementioned previous work in adolescent (PND 30–35) offspring (Titterness and Christie, 2012), in which females had potentiated LTP, it is unclear if the increase in glutamine synthetase is an adult phenomenon or also contributes to the adolescent phenotype. In adult (PND 55–70) non-PAE animals, depletion of intracellular glutathione mirrors PAE-associated declines in LTP in males, while also mirroring the lack of a decline in females (Patten et al., 2013). In PAE animals, increasing glutathione levels recovered the LTP deficits observed in males (Patten et al., 2013), further supporting this antioxidant’s role in regulating sex-specific, PAE-associated changes in hippocampal synaptic plasticity. Thus, hippocampal-dependent cognitive processes remain a promising area for future research on interactions between PAE and chromosomal sex, and sexually dimorphic vulnerability to early-adulthood cognitive deficits.

Additional research into the sex-specific impact of PAE on late adulthood cognitive function, and particularly cognitive decline, is warranted. Aging women experience higher rates of Alzheimer’s disease and dementia than men, which may be due to longer lifespan and lower biological ages, as shown by biomarker assessments (Hägg and Jylhävä, 2021). Data from self-report surveys of adults with FASDs suggest that early onset dementia is 209.3 times more likely in this population (Himmelreich et al., 2020), indicating that PAE may shift the natural onset and time-course of cognitive decline. A few recent studies have investigated the impact of PAE on Alzheimer’s disease in preclinical models. In the triple transgenic (3xTg-AD) Alzheimer’s disease mouse model, PAE offspring showed similar cognitive decline to control offspring in some domains, but also increased decline in performance on the novel object recognition task (Walter et al., 2022) and when navigating the Barnes maze at 4 months of age (Tousley et al., 2023). Sex differences were specifically uncovered in assessments of cued fear conditioning. Female 3xTg-AD PAE mice were hyperresponsive to cue presentation, exhibiting greater freezing response than female 3xTg-AD control exposure mice at 7 months, and greater freezing response than male 3xTg-AD PAE mice at 11 months (Walter et al., 2022). In the general population, there is a robust sex difference in cognitive function and decline, in which women exhibit greater cognitive reserve, but decline faster compared to age-matched men (Levine et al., 2021). Additional clinical and preclinical studies are needed to determine if these sex differences in cognitive decline occur with the same magnitude in individuals with PAE, and, in light of the self-report survey, if both sexes have a similarly advanced timeline of cognitive decline.

### 3.2. Neurobehavioral outcomes

#### 3.2.1. Adolescence

For individuals with PAE, cognitive impairments often co-occur with neurobehavioral disorders. Flannigan and colleagues estimate that collectively across the lifespan (ages 1–61), males with FASDs experience significantly higher rates of ADHD and conduct disorder than females, while females with FASDs have significantly higher rates of anxiety and mood disorder/depression than males (Flannigan et al., 2023). Young adolescents (mean age: ~14 years) and their parents have reported a multitude of behavioral problems associated with PAE, including antisocial and delinquent behaviors (Olson et al., 1997). Although the majority of sampled mothers self-reported alcohol consumption below binge-levels during pregnancy, estimated quantities of alcohol consumption positively correlated with adolescent behavioral problems, as did alcohol consumption earlier in pregnancy. The association between maternal drinking rates and negative adolescent experiences, including impairments in attention, arithmetic skills, and spatial-visual memory, was later reinforced in a longitudinal assessment of children with PAE (Streissguth, 2007). However, there is an important, sex-biased consideration in the interpretation of these maternal drinking rates and adolescent behavioral disorders. Within the general population, the onset of neuropsychological disorders in adolescents are notably distinct between boys/men and girls/women. For instance, the diagnoses of conduct disorder, autism spectrum disorder, and ADHD, are more prevalent in adolescent males than females among the general population (Zahn-Waxler et al., 2008; Costello et al., 2003). In contrast, females demonstrate more frequent onset of major depressive and generalized anxiety disorders during adolescence compared to males (Zahn-Waxler et al., 2008; Costello et al., 2003) and greater rates of burnout/stress-related disorders and eating disorders (Peper et al., 2020). Of particular consideration for adolescents with PAE are the data from the aforementioned study by May and colleagues (May et al., 2017) that found girls were more likely than boys to survive high levels of PAE, and therefore, may have higher rates of severe phenotypes for neurobehavioral outcomes that are associated with frequency and/or quantity of maternal alcohol use.

Although PAE has an established association with a range of neuropsychiatric disorders in adolescents, it remains unclear whether PAE: 1) is the sole cause of disorder development; 2) is a common covariate to other biological and environmental factors that contribute to disorder development; 3) exacerbates existing symptomology to increase the likelihood an individual is diagnosed; or 4) shifts the timing of disorder-onset to occur earlier in life. It is further unknown, at present, how PAE interacts with chromosomal and biological sex to influence symptom severity and onset. However, select research of adolescent psychological and neurological health highlight an interaction between PAE and sex in both clinical and preclinical assessments (Table 3).

A quarter of the 9,719 participants born between 2005 and 2008 and described in an Adolescent Brain Cognitive Development (ABCD) study (Lees et al., 2020) were exposed to alcohol during fetal development. Preadolescent children (9–10.9 years old) with PAE experienced significantly higher rates of separation anxiety disorder, specific phobias, ADHD and oppositional defiant disorder. These children also displayed attention deficits and altered sensation-seeking behavior, both of which were positively correlated with estimates of maternal drink quantities. Notably, being female was positively associated with behavioral scores and negatively associated with cognitive scores, indicating that females have greater behavioral problems and worse cognitive performance than males. Contrastingly, in a sample of 1,250 adolescents (mean age: 17 years), PAE corresponded with overall greater expression of conduct disorder symptoms compared to non-exposed controls, but symptoms were most prevalent in adolescent males compared to females (Disney et al., 2008). An additional study found that adolescents (mean age: 14 years 5 months) with PAE experience higher rates of behavioral problems related to conduct, delinquency, coping and stress, and substance use that persisted after including sex as a covariate, given that males displayed more of the problems assessed than females (Olson et al., 1997). However, others have found that sex as a biological variable does not interact with PAE to alter neurobehavioral outcomes in children (5–7 years) or adolescents (10–16 years) with a history of PAE (Panczakiewicz et al., 2016); rather, PAE, independent of sex, hinders their performance on a variety of neuropsychological and behavioral assessments. Thus, the interaction between sex and PAE is likely complex, and requires much more investigation during adolescence to draw definitive associations.

Preclinical investigations have further interrogated this interaction between biological sex, adolescent behavior, and corresponding neurophysiology. In Sprague-Dawley rats, a single period of ethanol exposure during gestation has been associated with generalized anxiety-like behavior across multiple assays in adolescent (PND 42–48) male offspring, without influencing adolescent females of the same litters (Rouzer et al., 2017). This behavior corresponded with sex-specific changes in GABAergic neurotransmission within the medial nucleus of the central amygdala, a hub of anxiety behavior regulation, and sex-specific expression and function of the stress peptide receptor, corticotropin releasing factor receptor 1, within the same sub-region (PND 40–8) (Rouzer and Diaz, 2022). This model of ethanol exposure has further corresponded with changes in offspring social preferences, which were age and sex-specific (Diaz et al., 2016). PAE males demonstrated distinct social deficits earlier in adolescence, at PND 28, than female counterparts, including decreased social investigation of an unfamiliar cage partner and decreased ratio of social preference to avoidance. Sex-specific deficits were also found in later adolescence (PND 42), with PAE females displaying a continued decrease in play behavior and similar decreases in social investigation and ratio of social preference to avoidance to age-matched males.

#### 3.2.2. Adulthood

Previous longitudinal studies indicate that mental health disorders such as depression and anxiety are overall more prevalent in adults with FASDs (Barr et al., 2006; Famy et al., 1998). In the general population, both depression and anxiety are more prevalent in females than in males, and females present with more severe depression symptoms and more somatic body symptoms of anxiety than men [reviewed in Altemus et al., 2014]. A few studies have investigated mental health in adults with PAE/FASDs and included sex as a biological variable. Two studies examined self-reported measures of social participation and adaptive functioning (Lynch et al., 2015), mental health, substance abuse (see Section 3.3), and issues with the legal system (Lynch et al., 2017) in young adults (~23 years old) in a longitudinal FASD cohort. These studies stratified participants based on the identification of FASD-related phenotypes, resulting in a comparison of individuals with: PAE with dysmorphology, PAE without dysmorphologies but with cognitive effects, PAE without dysmorphology and with no cognitive effects, and a control group matched by socioeconomic status with no disabilities. Significant interactions between sex and group were identified for adaptive functioning (Lynch et al., 2015), with cognitively-affected males displaying the lowest and cognitively-unaffected females displaying highest affective function scores. Intriguingly, the difference between these scores was larger than the differences between males and females in the control group, whereas there was little difference in scores between the sexes in the group with dysmorphology. Additional significant interactions were found for multiple outcomes associated with difficulties with the legal system, including total times arrested and charged, number of charges resulting in a conviction, and total time incarcerated, with males without cognitive effects reporting the highest values in all these groups (Lynch et al., 2017). These data mirror seminal work by Dr. Ann Streissguth that showed males with PAE have higher prevalence for trouble with the legal system across adolescence and adulthood, with being male and PAE-affected (with only some physical characteristics) increasing the odds of experiencing trouble with the law (Streissguth et al., 2004).

A more recent study examined mental health of older adults (mean age: ~38 years) (Coles et al., 2022). This study included sex as an exogenous factor in a path analysis, preventing a full gender-stratified assessment; however, they did identify that commonly occurring sex differences in mental health disorders are maintained for adults with PAE. Given that their study design was ~ 2:1 adults with PAE:socio-economic status-matched adults, if sex differences were diminished in adults with PAE, significance would have been more difficult to achieve. Yet, gender had indirect effects (through adverse childhood experiences) on depression diagnosis and severity, anxiety, and bipolar diagnosis (all associated with female gender) and direct effects on ADHD diagnosis (associated with male gender). Critically, PAE was directly associated with higher amounts of adverse childhood experiences (ACEs, see more in section 5), and the effects of PAE on depression, anxiety, and bipolar disorder were indirectly mediated by ACEs. These data indicate that while sex differences in the prevalence of mental health disorders are maintained for adults with PAE, the magnitude of the sex differences in incidence and/or severity may be exacerbated due to compounding effects of sex or gender and PAE. This hypothesis, however, requires further testing in additional studies balanced for sex-stratified assessments.

Preclinical studies have widely examined anxiety phenotypes in adulthood following PAE. A few studies examined social anxiety by measuring time spent interacting or avoiding a stranger rodent as one measure of social anxiety-like behavior. Adult PAE females interacted significantly less with a novel rat than PAE males [PND 120, Bake et al., 2021] and had significantly decreased social preference to avoidance ratios, indicative of social indifference, compared to their saline controls, with no differences between the male groups [PND 77, Diaz et al., 2016]. However, this female bias towards increased social anxiety-like behaviors following PAE may depend on the window of exposure and genetics, as work in a separate strain of rats found that both sexes showed social avoidance in adulthood (PND 75) when exposed during a period which overlapped with the previous two studies, and did not display any difference in social preference with an earlier exposure (Mooney and Varlinskaya, 2011). A similar female-bias towards anxiety was found for a novel feeding environment in which PAE females exhibited increased latency to consume food compared to control groups [PND 112–142, Hofmann et al., 2005]. Females, but not males, were overall responsive to the administration of an anxiolytic serotonin receptor 1A agonist to increase feeding behavior, which also diminished the feeding latency for PAE females. Additional anxiety-like behaviors have been examined using the elevated plus maze and open field tests, in which increased time avoiding more exposed areas of the apparatus indicates anxiety-like behavior. Contrasting effects of PAE have been identified for these tasks, as one study found a main effect of PAE to increase percent of time in the open arm of the elevated plus maze [P80, Diaz et al., 2016], another found a main effect of PAE to decrease the amount of time spent in the exposed center of the open field [PND 120, Bake et al., 2021], and another found that PAE females, but not males, were less likely to spend time in the center of the open field than their respective controls [PND 56–63, Wang et al., 2023]. Furthermore, exposure to stress may result in sex-specific increases in anxiety-like phenotypes in PAE adults. For the elevated plus maze, following chronic mild stress in adulthood (PND 60–90), PAE males spent less time in the open arm than control males, while there were no differences between PAE and control females (Hellemans et al., 2008). In the open field test, PAE males only showed decreased time spent in the center compared to controls following acute restraint stress, while PAE females were no longer significantly different than their controls following acute restraint stress. PAE males similarly displayed increased center avoidance compared to controls, but only after an acute restraint stress [PND 56–63, Wang et al., 2023].

### 3.3. Alcohol use in adolescence and adulthood

PAE has been associated with increases in substance dependence and misuse in clinical investigations (Olson et al., 1997), with misuse reports beginning at the age of 14 and continuing into young adulthood (Streissguth, 2007). Notably, PAE specifically, rather than a family’s history of alcohol misuse, predicted problematic drinking in 14-year-old adolescents (Baer et al., 1998). Young adults (21 years old) with PAE demonstrated increased scores on the Alcohol Dependence Scale (Baer et al., 2003). For young adults (22 years old), higher levels of PAE in either the first or third trimester was associated with greater odds of having two or more symptoms of alcohol misuse and dependence, which indicates a higher risk for later life alcohol use disorders (Goldschmidt et al., 2019). Being male was associated with greater odds of having two or more symptoms of alcohol misuse and dependence, and with heavier patterns of drinking. Although the interaction of sex and PAE was not examined, these data preliminarily suggest that males with higher PAE may be the most at risk for alcohol misuse. However, the number of studies investigating the effects of PAE on offspring alcohol consumption and preference are limited in their explicit investigation of sex as a biological variable [Table 4, reviewed in Chotro et al., 2007].

While overall investigations of adolescent drinking behaviors in preclinical PAE models have reported variable and sex-specific outcomes [reviewed in Gaztañaga et al., 2020], a few studies have reported that PAE males, but not females, demonstrate greater preference for ethanol during childhood and adolescence than control offspring [PND 25–16 mice, Randall et al., 1983; PND 33–38 rats, (Pepino et al., 2004]. An additional study of PAE-induced drinking in Sprague-Dawley rats found decreased aversion to alcohol in PAE males in early adolescence (PND 26–31) (Gore-Langton and Spear, 2019). PAE females in late adolescence (PND 34–55), using a three-bottle-choice paradigm, showed decreased intake and preference for lower concentration (5%) alcohol, with no changes in preference for higher concentration (10%) alcohol. In contrast, age-matched PAE males demonstrated no change in preference from controls at either concentration. However, for adult (PND 56–81) PAE males in this study, preference and intake for lower concentration alcohol were increased and overall intake of higher concentration alcohol was decreased. A number of factors may contribute to this age discrepancy in PAE male alcohol consumption, potentially including an interaction between gonadal hormones and addictive behavior. A recent publication characterizing ethanol preference using the Four Core Genotypes mouse model (Sneddon et al., 2023), a tool to investigate the separate contributions of sex chromosomes and gonadal hormones to sexually dimorphic characteristics and behaviors (De Vries et al., 2002), revealed that XY chromosome pairs were associated with greater preference for 15% ethanol, while subjects with testes exhibited less aversion to quinine adulteration [≥PND 60; Sneddon et al., 2023]. This may indicate that, in non-PAE offspring, both chromosomal composition and hormone production may contribute to ethanol preference in males.

In contrast, clinical data suggest that PAE is associated with increased drinking by women during adolescence and decreased drinking over the lifespan by male offspring (Griesler and Kandel, 1998), and that in adulthood, a larger percent of females with PAE experience substance abuse problems than males (70% and 53%, respectively) (Streissguth et al., 1996). However, more recent work examining young adults (~23-years-old) has shown that proclivity for drinking may vary by the constellation of domains impacted by PAE, with larger effects seen in males (Lynch et al., 2017). In this aforementioned study (see Section 3.2.2), individuals were stratified by domains impacted by PAE (e.g. PAE with FASD dysmorphology, PAE and cognitively affected, PAE and cognitively unaffected). Both PAE group and sex influenced self-reported current amount of alcohol used and blood concentrations of alcohol biomarker gamma glutamyl transferase (GGT). or blood levels of GGT, males in the PAE, cognitively unaffected group were > 1.75 times that of other PAE groups and the socioeconomic status-matched control group, whereas females had similar levels of GGT across all groups. For self-reported current amount of alcohol used, males in the PAE, cognitively unaffected group reported > 2 times the amount of absolute alcohol equivalents per week compared to the other PAE groups and > 4 times the amount of alcohol per week as the control group. Females in the PAE, cognitively unaffected group also reported elevated levels of current alcohol use, with > 1.3 times the average amount per week of the other PAE groups and 3 times the amount of the control group. In consideration of this discrepancy, preclinical models suggest the dose of ethanol during gestation may interact with sex to result in sex-specific increases in adolescent ethanol consumption. For example, intragastric administration of 1 g/kg ethanol to dams during the final three days of pregnancy increased pre-weaning (PND 14) female ethanol consumption compared to non-exposed females, without impacting males (Chotro and Arias, 2003). In contrast, at a higher dose of maternal exposure (2 g/kg ethanol), pre-weaning males exhibit more ethanol consumption than non-exposed controls, whereas female consumption was not different from sex-matched controls. Additionally, social isolation during adolescence (from PND 21 through testing) augmented offspring ethanol self-administration in a two-bottle choice paradigm, with the highest rates of drinking occurring in offspring (PND 56–84) with a history of PAE and social isolation (Fernández et al., 2019). Unfortunately, this experiment was limited to male subjects, preventing investigation of sex as a biological variable. Together, these studies highlight a multitude of environmental factors that influence PAE-induced drinking differences between male and female adolescent offspring [reviewed in Varlinskaya and Spear, 2015].

Ethanol self-administration in young adult (PND 70–81) rat offspring was assessed concurrently with the studies on social interaction outlined above [see Section 3.2.2, (Diaz et al., 2020)]. While PAE increased ethanol consumption in both sexes, PAE males who demonstrated greater social anxiety also drank the most of all male subjects. In contrast, PAE did not influence ethanol consumption in females, regardless of social anxiety behavior. Social environment also modified alcohol consumption in PAE offspring in a sex-dependent manner. For example, for saline-treated (control) males, drinking in isolation increased alcohol consumption levels, while social anxiety did not have an effect. This was distinct from PAE males, for whom high social anxiety increased drinking behavior, but drinking context (alone compared to a social setting) did not have an effect. For saline-treated females, high anxiety in a social setting increased drinking behavior, while isolation decreased alcohol consumption levels. Again, this was distinct from the sex-matched PAE offspring, whose drinking behavior was not impacted by either anxiety or social environment. In young adult Wistar rats (PND 60), PAE was associated with increased voluntary alcohol consumption in a two-bottle choice paradigm in male offspring compared to sex-matched control offspring, but not in female offspring (Kokhan et al., 2022). In this paradigm, female offspring consumed more alcohol overall than male offspring; therefore, the lack of a PAE effect in females may be due to a “ceiling effect,” in that control females may already be consuming at the height of their tolerance.

## 4. Sex differences in PAE outcomes with age-related assessments/vulnerability

### 4.1. Adolescence: Brain structure

Although the human brain reaches full size by the early postnatal period, adolescence serves as a significant window of restructuring and synaptic pruning, in which myelination occurs in an inferior-superior and poster-anterior direction (Lenroot and Giedd, 2006). This is particularly important for understanding behavior and cognition in adolescents, as areas responsible for behavioral control, decision-making and risk assessment are still maturing for most of this age span (Konrad et al., 2013). Importantly, males and females differ in their neurodevelopmental timelines, in part due to programming by sex steroids which alter the onset of puberty [reviewed in Sisk and Foster, 2004]. As an organ with prominent steroid receptor expression, the adolescent brain is highly susceptible to hormone concentrations, which can contribute to between and within-sex variability for neural maturation and restructuring (Sisk and Zehr, 2005). Furthermore, when linking brain function and structure with behavioral measures, normally-developing children and adolescents experience sex-specific differences in the strength of the association between brain imaging and subject measures, such as IQ (Lange et al., 2010). Thus, comparisons of neural composition in adolescents should not be treated as predictors of consistent behaviors between boys and girls.

In general, the primary PAE-characteristic behavioral and cognitive impairments are believed to be associated with delayed or impaired brain development, mainly reduced cortical volume, defective corpus callosum integrity, and an underdeveloped cerebellum [Kane et al., 2021; systematically reviewed in Norman et al., 2009], along with abnormal hippocampal development (Willoughby et al., 2008). Brain imaging studies of adolescents have provided great insight into PAE-induced alterations to brain structure, including distinctive neural activity and structural composition during early development (Lees et al., 2020; Biffen et al., 2022; Donald et al., 2015) [reviewed in Lebel et al., 2011]. Unfortunately, many existing imaging studies, particularly those with young adults, exhibit the same limitations previously addressed for investigating sex as a biological variable (Table 5): namely, sample sizes too small to sufficiently power analyses of sex differences [Archibald et al., 2001; Sowell et al., 2001; Astley et al., 2009; Cortese et al., 2006; Li et al., 2008, to name a few], even when males and females are equally represented in participant pools. Far more research is necessary to define the interactions between PAE and sex in brain structure and function during this critical period of neurodevelopment.

A few studies have investigated sex differences in preadolescent children and adolescents with FASD. Functional magnetic resonance imaging (fMRI) uncovered hypoconnectivity between the auditory network and the right ventral diencephalon in preadolescent children (9–10.9 years old) with a history of PAE, who concurrently exhibited hyperconnectivity between sensorimotor and salience networks (Lees et al., 2020). Importantly, there were significant associations between chromosomal sex and offspring measurements in this cohort, including sexually-dimorphic structural and resting-state fMRI metrics. Although an interaction between sex and PAE was not explicitly addressed, both PAE and being female were significantly associated with greater cortical volume. Sex- and region-specific changes in white matter volume have been reported in a separate investigation of children/adolescents with FASD (ages 9–16) (Uban et al., 2017). White matter tracts were measured with diffusion tensor imaging, and revealed that boys with FASD exhibited more white matter in the colossal body, cingulum, corticospinal tract, inferior fronto-occipital fasciculus and superior longitudinal fasciculus than controls. These PAE-associated changes in white matter microstructure were not similarly observed in girls with FASDs; rather, PAE was associated with a trend toward less white matter in the inferior fronto-occipital fasciculus and uncinate fasciculus compared to sex-matched controls. Notably, associations between hormone levels and white matter were different between males and females, *and* between control and PAE subjects, indicating that sex-specific outcomes of PAE may be associated with dysregulated neuroendocrine activity.

In an aforementioned study by Carter and colleagues [see Section 3.1.1, Carter et al., 2014], the importance of hormonal contributions to FASD symptoms was further supported by an investigation of 14-year-old adolescents with PAE. Both boys and girls with PAE exhibited elevated testosterone levels compared to unexposed controls, with increasing self-reported levels of maternal alcohol exposure corresponding to increased testosterone production in both sexes; however, with higher levels of PAE there was a diminshed relationship between testosterone and the pubertal phenotype of pubic hair development. Altered sex hormone levels and/or signaling may directly impact brain development, as testosterone levels during adolescence are positively associated with amygdalar gray matter in both sexes, hippocampal size in females, and diencephalic structures in males (Neufang et al., 2009). In older adolescents/young adults (~22–23 years old) with a history of PAE, males and females exhibit different degrees of PAE-associated deficit across a multitude of cortical structures, with PAE males demonstrating greater volumetric reductions than PAE females overall (Chen et al., 2012). This male-biased growth deficit in gray matter volume was similarly observed in younger subjects (children and adolescents, 6–17 years old) with FASD (Nardelli et al., 2011), which the researchers posited could be associated with reduced testosterone, which has been noted in preclinical models, but could instead be associated with decreased testosterone signaling.

### 4.2. Adulthood: Inflammation and cardiovascular disease (CVD)

Sex differences in immune function and inflammation occur in the general population with aging. For example, the preponderance of autoimmune disorders occur in females, while males experience higher risk for malignancy in non-reproductive cancers [reviewed in Klein and Flanagan, 2016]. The rates of CVD risk factors assessed in adults from 2011 to 2015 showed women that, compared to men, exhibit increased body mass index, marginal reduction in total cholesterol, poor control of hypertension and diabetes mellitus, and increased dyslipidemia (Peters et al., 2019), overall indicating that females experience a greater burden of cardiovascular risk factors that also contribute to a pro-inflammatory milieu. These sex differences in adulthood are multifactorial, with a complex interplay of genetics, hormones, and environmental factors, including PAE.

There is a wealth of information available on the impact of PAE on the immune and endocrine systems during fetal life, and the interactions between both systems are critical for normal brain development and the long-term health of offspring. However, fewer studies have addressed the sex-specific regulation of the endocrine and immune system in adults with FASDs (Table 6). The cross-talk between endocrine/metabolic and immune organs plays a crucial role in inflammatory responses in adulthood. In this review, we refrain from detailing the sex-specific consequences of PAE on adult metabolic and endocrine systems, as it is discussed elegantly in another chapter of this issue by Smith, Mooney, and colleagues. We will focus exclusively on adults with FASDs, summarize the available evidence on PAE and the immune system, and emphasize the role of biological sex in the modulation of inflammatory responses.

Adults with FASDs are reported to have high rates of ear infections, i.e. 147 times more than the general population, suggesting a weakened immune system and persistent inflammation (Himmelreich et al., 2020). Interestingly, studies have reported a higher prevalence of autoimmune diseases in adults with FASDs, indicating a hyperreactive immune status (Noor and Milligan, 2018; Terasaki and Schwarz, 2016). Moreover, these findings suggest that alcohol exposure during early development persistently alters the tolerance of immune cells to self-antigens, which is known to have alarmingly dangerous consequences. These PAE-mediated deficits are intricate and most often become apparent only after a second hit of other life adversities, such as physiological stress (see Section 5) or other immune challenges later in life. Alcohol exposure during pregnancy is known to elevate inflammatory cytokines in maternal circulation (Bodnar et al., 2018), and can elevate inflammatory cytokines in both children with PAE (Fiore et al., 2022; Bodnar et al., 2020) and adults with FASDs (Himmelreich et al., 2020), as evidenced by self-reported higher incidence of comorbid conditions associated with inflammation. A preclinical study on PAE throughout gestation and long-term effects on the immune system show that adult male rats (3 months of age) have decreased the number of thymic cells and reduced proliferation when exposed to the T-cell mitogen, concanavalin A (Weinberg and Jerrells, 1991). Recently, in a rat PAE model, sexually dimorphic peripheral inflammatory responses were noted in 5-month-old adults. For example, adult females exhibited greater signs of peripheral inflammation, with increased levels of pro-inflammatory cytokines in the liver and mesenteric adipose tissue; however, PAE males had lower levels of cytokines, both pro- and anti-inflammatory, suggesting impaired immune system function (Bake et al., 2021). This may be due to the poor reserve of cells in immune organs, such as the spleen and thymus, in young males, as reported in the aforementioned study by Weinberg and Jerells (Weinberg and Jerrells, 1991). Additionally, the severity of adjuvant-induced rheumatoid arthritis was greater in adult female Sprague-Dawley rats exposed to PAE throughout gestation and up to weaning, and the addition of chronic mild stress during adolescence (PND 31–41) significantly worsened the outcome of the recovery. When adolescent stress was followed by injection of Freund’s adjuvant in young adulthood (PND 55–60), PAE females had elevated corticosterone levels, faster arthritis progression, and delayed recovery (Bodnar et al., 2022). This delayed recovery occurred alongside increased total number of macrophages present at the inflamed joint in PAE and chronic mild stress-females, suggesting that in addition to the hyperresponsive HPA axis, the heightened immune response in PAE females contributed to the delayed resolution of arthritis. Of note, these kinds of persistent, PAE-associated immune alterations increase the likelihood of other adult-onset diseases, including CVD and neurological disorders.

CVD, primarily ischemic heart disease and stroke, remains the leading cause of death and disability worldwide, leading to > 20 million deaths in 2021 (Vaduganathan et al., 2022). In addition to well-known risk factors, such as age and biological sex, early life experiences are important modifiers of CVD (Elford et al., 1991; Parkington et al., 2010; Winning et al., 2016). The majority of FASDs are silent phenotypes, but have serious long-term consequences for health (Ipsiroglu et al., 2013; Mattson and Riley, 2011; Popova et al., 2023), including risk factors for developing CVD. A recent retrospective chart review (Weeks et al., 2020) has shown that for adults with FASDs (median age: ~30 years), females are significantly more likely to be overweight/obese compared to age, sex, and race/ethnicity-matched controls, while males are significantly more likely to be underweight. Additionally, males with FASDs were significantly more likely to have lower high-density lipo-protein cholesterol levels, elevated triglycerides, and greater rates of type 2 diabetes than control males.

PAE can have lifelong consequences for cardiovascular health and function. Preclinically, PAE resulted in increased activation of caspase-3 in ventricular cardiomyocytes from 4-month-old adult male PAE rats (Ren et al., 2002), suggesting that PAE increases apoptosis of heart cells in adult offspring. Although only male rats were examined in this study, another study reported that PAE causes structural damage in the heart of both males and females. For example, offspring born to pregnant rats fed a 6% w/v diet throughout gestation displayed hypertrophy of the ventricular wall in both males and females at 8 months of age (Nguyen et al., 2014). While both males and females may be at risk for altered cardiac structure, functional outcomes such as blood flow may also be altered in an age- and/or sex-specific manner. In a mouse model of PAE, moderate alcohol exposure altered cranially-directed blood flow in an age-dependent manner, i.e., a hypertensive phenotype in the carotid arteries of young adults (3 months old) and a hypotensive pattern in mature (6 months old) and middle-aged animals (12 months old) (Bake et al., 2017). Interestingly, while control animals exhibited a sex difference in the acceleration of blood flow in the carotid artery at 6 months of age, with females displaying greater acceleration, there were no effects of sex on carotid blood flow in PAE animals. Another study showed that low levels of PAE resulted in a hypotensive response to mild restraint stress in middle-aged (12 moths old) female, but not male, rats (Walton et al., 2019), indicating that middle-aged females may be more sensitive to stress-induced disruptions to vascular tone. These kinds of blood flow alterations to vital organs like the heart and brain could alter the vascular physiology and render PAE adults more vulnerable to adult-onset diseases, such as heart diseases and stroke. Correspondingly, it is not surprising that adults with FASDs self-report 21.1 times higher rates of cardiomyopathy, 1.6 times higher rates of coronary heart disease, and 1.9 times higher rates of hypertension than the general population (Himmelreich et al., 2020).

A contributing factor to sex differences in CVD following PAE may include altered vascular responsivity to vasodilators and vasoconstrictors. In prenatal alcohol-exposed adult sheep, both sexes demonstrated increased vasodilatory response of cerebral arterioles to neuronal vasoactive intestinal peptide, although sex was not included as a variable in statistical analyses (Ngai et al., 2008). In contrast, while stimulation of nitric oxide synthase typically dilates vasculature, another study showed that infusions of endothelial and neuronal nitric oxide synthesis agonists, ADP and NMDA respectively, reduced the dilation of cerebral arterioles in 3–4-month-old PAE rats (Cananzi and Mayhan, 2019). Surprisingly, the infusion of vasoconstrictor angiotensin II resulted in significantly increased vasodilation in cerebral arterioles of adult (3.5–4 months old) PAE female rats compared to age and sex-matched control rats (Saha et al., 2021). This effect of PAE was not found during adolescence (1–1.5 month old), nor did adult male PAE rats experience a significant increase in vasodilation following angiotensin II infusion compared to age and sex-matched control rats. These studies suggest that cerebrovascular responses to vasoactive molecules are modified by PAE, altering neurovascular coupling and blood flow regulation in adults, and that the effects of PAE on adult blood flow may be sex-specific.

Ischemic and hemorrhagic stroke are the 2nd and 3rd largest contributors to CVD-associated mortality, respectively (Vaduganathan et al., 2022). Collectively, they contributed to 36% of worldwide CVD-associated deaths in 2021 (Vaduganathan et al., 2022). Both clinical and pre-clinical studies show strong evidence that PAE and sex interact to influence metabolic diseases in adulthood [reviewed in Akison et al., 2019] (Weeks et al., 2020). Notably, metabolic disorders and altered neurovascular coupling are important risk factors for stroke. Recent preclinical studies revealed that prenatal alcohol modifies ischemic stroke outcomes in adults. For example, adult male and female PAE mice (3 months old), when subjected to experimental ischemia via intra-luminal blockage of the middle cerebral artery, had poor neurological recovery in the acute stage (Bake et al., 2017). However, PAE females exhibited greater deficits in sensorimotor performance after stroke, as measured by the adhesive removal test, compared to PAE males. In a more recent study, endothelin-1-induced stroke caused worse neurological deficits and sensorimotor integration in the acute stage in of PAE rats (5 months old), an effect that was sex-independent (Bake et al., 2022). Additionally, this study reported that long-term stroke outcomes in PAE-rats are modified by sex. PAE males displayed earlier onset of stroke-induced deficits in social behavior than control males, while exhibiting spatial learning deficits, including increased latency to reach the goalbox in a Barnes maze platform. However, in PAE females, the behavioral domain that was modified by stroke was associative learning in the fear conditioning test. Critically, existing therapeutics for stroke already display sex differences in efficacy [reviewed in Sohrabji et al., 2017], and these therapies may be further modified in prenatally alcohol-exposed individuals.

## 5. Additional variables of consideration for future FASD research

At first glance, understanding the impact of genetic sex on lifelong outcomes following PAE appears to be relatively straightforward, requiring a balance of samples by sex and disaggregation of clinical and preclinical data by sex, including the generation of effect sizes to allow for larger follow-up analyses (Denfeld et al., 2022). However, while chromosomal sex alone can contribute to health outcomes, numerous sex-related variables, including lived experience, environment, and developmental age, could further contribute to sex-biased outcomes (DiMarco et al., 2022). Furthermore, common co-occurring factors can confound the expression of PAE’s effects between male and female offspring.

One such factor is co-exposure to substances other than alcohol. Polysubstance use in individuals of childbearing age is common. An analysis of the National Survey on Drug Use and Health data from 2006 to 2014 found that 23.9% of non-pregnant women and 5.1% of pregnant women (18–49 years old) reported engaging in polysubstance use, with 18–25 years old reporting the highest prevalence of substance co-use (60.3%). In particular, users of alcohol commonly co-use marijuana (Subbaraman and Kerr, 2015), opioids (Cicero et al., 2020), and nicotine (Qato et al., 2020). Although research investigating prenatal polysubstance use and sex-specific outcomes is currently very minimal, studies investigating prenatal polysubstance exposure of alcohol and cannabinoids have reported sex-specific outcomes in offspring behavior that are not observed in offspring exposed to a single substance prenatally. For instance, prenatal co-exposure impaired motor coordination in female, but not in male, juvenile offspring (Breit et al., 2019), a phenotype that was observed in male offspring exposed to alcohol alone. In adolescent male rats, co-exposure to alcohol and tetrahydrocannabinol throughout gestation resulted in greater motor activity and time spent in the center of an open-field apparatus compared to non-exposed controls, a phenotype that was absent in female littermates (Breit et al., 2022). Co-exposure has further produced distinct, sex-specific outcomes in spatial learning and memory compared to offspring with single-drug exposure (Lei et al., 2023). Importantly, all of these commonly consumed substances have been independently associated with sex-specific offspring health outcomes following prenatal exposure [see reviews of: prenatal opioid exposure Terasaki et al., 2016; prenatal opioid exposure Simmons et al., 2023; prenatal nicotine/tobacco exposure Sikic et al., 2022; prenatal cannabinoid exposure Tirado-Muñoz et al., 2020], and there are a multitude of common neurobehavioral domains that are targeted by these substances, including attention and hyperactivity, cognitive performance, and increased drug-seeking behaviors (Terasaki et al., 2016; Lowe et al., 2022; Rouzer et al., 2023; Duko et al., 2022). Thus, understanding how these substances interact prenatally, and whether the outcomes on offspring health are sexually dimorphic, will better inform targeted treatment in translationally relevant models of prenatal exposure.

Another common phenomenon observed in children with PAE is early-life stress. Stress is a critical contributor to negative, sex-biased outcomes following PAE, sensitizing the HPA axis and leading to potentiated and maladaptive stress responses that increase risk for other secondary conditions, including anxiety and depression [reviewed in Weinberg et al., 2008; Hellemans et al., 2010]. Although several studies have demonstrated a clear relationship between PAE and increased rates of adverse childhood experiences (ACEs) and/or environmental stressors (Flannigan et al., 2023; Coles et al., 2022; Uban et al., 2020; Kambeitz et al., 2019), these studies either did not stratify outcomes by sex, or lacked non-PAE control subjects to determine whether PAE produces sex-specific outcomes that are distinct from the general population. Preclinical PAE models that include ACEs-like childhood stress have reported relationships between PAE, ACEs, and sex-specific anxiety-like phenotypes. In one preclinical study, PAE was associated with reduced time spent in the open arms of the elevated plus maze, indicative of greater levels of anxiety, in young adult male, but not female, rat offspring (Hellemans et al., 2008). Male offspring who experienced PAE with subsequent chronic mild stress during adolescence exhibited an augmented reduction in open arm entries on this maze, while stress did not affect open arm entries in female offspring with PAE. A similar study examined PAE throughout gestation, combined with chronic unpredictable stress in early adulthood (Lam et al., 2019). In this study, adult male PAE offspring also displayed increased anxiety-like behavior, with decreased time in the center of an open field assay, while female PAE offspring exhibited decreased anxiety-like behavior, spending more time in the center of the open field assay and more often entering the open arms of an elevated plus maze. This anxiolytic-like effect of PAE on adult females was notably abrogated with chronic unpredictable stress. The distinct effects of prenatal stress with PAE have also been demonstrated in electrophysiological assessments of adolescent hippocampal activity, with co-exposure to stress blocking the enhanced LTP observed in unstressed PAE females (Titterness and Christie, 2012). Meanwhile, prenatal stress produced no significant change in hippocampal LTP levels in PAE males. Together, these findings show that early-life stress augments PAE-associated deficits and highlight the need for additional research that statistically accounts for sex as a biological variable.

Overall, the impact of sex on offspring health is multifactorial and additional sex-biased risk factors that contribute to negative health outcomes following PAE require additional investigation.

## 6. Conclusions

As discussed, PAE has pronounced impacts on health throughout the lifespan. During fetal life, PAE results in sex-specific effects on viability, growth, placental function, and cellular programming. Adolescents with PAE/FASDs experience distinct changes in their affect, mental health, and cognitive abilities, as well as in underlying neural structures and function. These kinds of disorders can persist into adulthood, or alternatively, prime developing systems for additional deficits in adulthood. In adults, PAE can increase risk for CVD, inflammation, and cognitive decline. There are numerous domains that require more investigation to define the sex-biased effects of PAE, including the separate impact of gender identity as a modifier of sexual dimorphism. Further investigations are also needed to better understand risk factors, which may themselves present in gendered or sex-specific manners, that worsen outcomes following PAE. This knowledge will not only identify windows and targets for intervention, such as early life stress, but allow for new treatment designs to improve outcomes for individuals with PAE of both sexes and across the continuum of gender.

## Figures and Tables

**Table 1:** Placental genes and proteins affected by PAE.

Gene/Protein	Reference	Species (age)	Exposure model	Expression (PAE v. Control)
*ANXA4*/ANXA4 Annexin A4	(Holbrook et al., 2019)	Human (GW > 37)		↓protein
	(Savage et al., 2020)	Rat (GD 20)	GD 1—20, voluntary consumption	↑protein
*FLT1*/VGFR1 Vascular endothelial growth factor receptor 1	(Holbrook et al., 2019)	Human (GW > 37)		↓protein (ns)
	(Lecuyer et al., 2017)	Human (GW 35—42)		↔protein
	(Lecuyer et al., 2017)	Mouse (GD 20)	GD 13–19, subcutaneous injection	↓protein
	^a^(Gårdebjer et al., 2014)	*Rat (GD 20)*	*GD [−4]−4, liquid diet* ^1^	↔*mRNA (labyrinth zone)*
*HSD11B1*/DHI1 (11β-HSD2) 11-beta-hydroxysteroid dehydrogenase 1	(Liang et al., 2011)	Mouse (GD17)	GD 11–17, gavage	↓mRNA
	^a^(Gårdebjer et al., 2014)	*Rat (GD 20)*	*GD [−4]−4, liquid diet* ^1^	↑*mRNA (labyrinth zone)*
	^a^(Lan et al., 2017)	*Rat (GD 21)*	*GD1 - GD21, liquid diet*	↑*protein*
	^b^(Wilcoxon et al., 2003)	**Rat (GD 21)**	**GD 8–21, liquid diet**	**↓female ↑male mRNA**
*IGF2*/IGF2 Insulin like growth factor 2	(Carter et al., 2018)	Human (GW mean 39)		↑mRNA (fetoplacental compartment)
	(Joya et al., 2015)	Human (GW 35–41)		↔protein
	(Joya et al., 2015)	Human trophoblast cell lines		↑protein
	(Downing et al., 2011)	Mouse (GD 9)	GD 9, gavage	↓mRNA
	^a^(Marjonen et al., 2018)	*Mouse (GD 9.5)*	*GD 0.5–8.5, voluntary consumption*	↔*mRNA*
	^a^(Kalisch-Smith et al., 2019)	*Rat (GD 13)*	*GD [−4]−4, liquid diet* ^1^	↔*mRNA*
	^a^(Marjonen et al., 2018)	*Mouse (GD 16.5)*	*GD 0.5–8.5, voluntary consumption*	↓*mRNA*
	^a^(Gårdebjer et al., 2014)	*Rat (GD 20)*	*GD [−4]−4, liquid diet* ^1^	↔*mRNA (labyrinth zone)*
	^a^(Gårdebjer et al., 2014)	*Rat (GD 20)*	*GD [−4]−4, liquid diet* ^1^	↑*mRNA (junctional zone)*
	(Shukla et al., 2011)	Rat (GD 21)	GD 8–21, liquid diet	↑mRNA^2^
*KDR*/VGFR2 Vascular endothelial growth factor receptor 2	(Holbrook et al., 2019)	Human (GW > 37)		↓protein
	(Lecuyer et al., 2017)	Human (GW 35 to 42)		↔protein
	(Lecuyer et al., 2017)	Mouse (GD 20)	GD 13–19, subcutaneous injection	↓protein
	^b^(Gårdebjer et al., 2014)	**Rat (GD 20)**	**GD [−4]−4, liquid diet** ^1^	**↑female mRNA (labyrinth zone)**
*PDCD10*/PDC10 (CCM3) Programmed cell death 10	(Holbrook et al., 2019)	Human (GW > 37)		↓protein (ns)
	(Savage et al., 2020)	Rat (GD 20)	GD 1—20, voluntary consumption	↓protein
*PGF*/*PLGF* Placental growth factor	(Lecuyer et al., 2017)	Human (GW 35–42)		↓protein
	(Lecuyer et al., 2017)	Mouse (GD20)	GD 13–19, subcutaneous injection	↓protein (labyrinth zone and whole placenta)
	(Rosenberg et al., 2010)	Rat (GD 20)	GD 1–20, voluntary consumption	↓mRNA
*PHLDA2*/PHLA2 Pleckstrin homology-like domain family A member 2	^b^(Kalisch-Smith et al., 2019)	**Rat (GD 13)**	**GD [−4]−4, liquid diet** ^1^	**↓female mRNA**
	(Shukla et al., 2011)	Rat (GD 21)	GD 8–21, liquid diet	↑mRNA^2^
*SLC2A1*/GTR1 (GLUT1) Solute carrier family 2, facilitated glucose transporter member 1	^a^(Kalisch-Smith et al., 2019)	*Rat (GD 13)*	*GD [−4]−4, liquid diet* ^1^	↔*mRNA*
	^b^(Kalisch-Smith et al., 2019)	**Rat (GD 15)**	**GD [−4]−4, liquid diet** ^1^	**↑female mRNA (labyrinth zone)**
	^a^(Gårdebjer et al., 2014)	*Rat (GD 20)*	*GD [−4]−4, liquid diet* ^1^	**↔mRNA (labyrinth zone)**
	^a^(Gårdebjer et al., 2014)	*Rat (GD 20)*	*GD [−4]−4, liquid diet* ^1^	*↔mRNA (junctional zone)*
*SLC2A3*/GTR3 (GLUT3) Solute Carrier family 2, facilitated glucose transporter member 3	^b^(Kalisch-Smith et al., 2019)	**Rat (GD 13)**	**GD [−4]−4, liquid diet** ^1^	**↓female mRNA**
	^b^(Kalisch-Smith et al., 2019)	**Rat (GD 15)**	**GD [−4]−4, liquid diet** ^1^	**↓male ↑female mRNA (labyrinth zone)**
	^a^(Gårdebjer et al., 2014)	*Rat (GD 20)*	*GD [−4]−4, liquid diet* ^1^	↔*mRNA (labyrinth zone)*
	^b^(Gårdebjer et al., 2014)	**Rat (GD 20)**	**GD [−4]−4, liquid diet** ^1^	**↓male ↑female mRNA (junctional zone)**
*TNF*/TNFA (TNF-α) Tumor necrosis factor	(Holbrook et al., 2019)	Human (GW > 37)		↑protein (ns)
	(Ruyak et al., 2022)	Human (GW mean 38–39)		↓mRNA
	(Ruyak et al., 2022)	Human (GW mean 38–39)		↔protein
	^a^(Terasaki and Schwarz, 2016)	*Rat (GD 17)*	*GD 10-GD 16, gavage*	↔*mRNA*
*VEGFA*/VEGFA Vascular endothelial growth factor A	(Lecuyer et al., 2017)	Human (GW 35–42)		↓protein (ns)
	(Holbrook et al., 2019)	Human (GW > 37)		↔protein
	(Lecuyer et al., 2017)	Mouse (GD 20)	GD 13–19, subcutaneous injection	↔protein
	^a^(Gårdebjer et al., 2014)	*Rat (GD 20)*	*GD [−4]−4, liquid diet* ^1^	↓*mRNA (labyrinth zone)*
*TNF*/TNFA (TNF-α) Tumor necrosis factor	(Holbrook et al., 2019)	Human (GW > 37)		↑protein (ns)
	(Ruyak et al., 2022)	Human (GW mean 38–39)		↓mRNA
	(Ruyak et al., 2022)	Human (GW mean 38–39)		↔protein
	^a^(Terasaki and Schwarz, 2016)	*Rat (GD 17)*	*GD 10-GD 16, gavage*	↔*mRNA*
*Other genes with noted sex differences*				
*RLIM*/RNF12 E3 ubiquitin-protein ligase RLIM	^b^(Kalisch-Smith et al., 2019)	**Rat (GD 15)**	**GD [−4]−4, liquid diet** ^1^	**↓mRNA (female only expression)**
Tet methylcytosine dioxygenase 3 *XIST*	^b^(Pinson et al., 2022)	**Mouse (GD 18)**	**GD 10, gavage**	↑**male mRNA**
X inactive specific transcript	^b^(Kalisch-Smith et al., 2019)	**Rat (GD 13)**	**GD [−4]−4, liquid diet** ^1^	**↓mRNA (female only expression)**

Placental genes and proteins affected by PAE in multiple studies. Genes altered by PAE in a sex-specific manner are also shown. Gene/protein names are from GeneCards and Uniprot, respectively. Names shown in parentheses indicate the name commonly used across the literature. Data were derived from whole placenta unless otherwise noted.

1 In this periconceptional model, exposure begins 4 days prior to mating and continues for 4 days after mating.

2 Significantly changed from control, but not pair fed, therefore expression change may be due to decreased caloric intake in PAE group.

a Studies in which sex differences were examined but no sex differences were identified (also denoted by italics).

b Studies that found significant differences in the effects of PAE that differed by sex (also denoted in bold).

GW, gestational week; GD, gestational day; ns, non-significant change in expression.

**Table 2:** Referenced cognitive performance studies investigating PAE and including both sexes, ranked and sorted by rigor in addressing sex as a biological variable.

Rigor Score^1^	Reference	Species (Age)	Includes Both Sexes	Main Effect of Sex	PAE by Sex Interaction	Statistics Performed	Strengths	Limitations
5	(An and Zhang, 2013)	Rats (Adolescent)	Yes	Yes	Yes	Multivariate ANOVA	Sex assessed statistically, appropriate controls included	
5	(Bake et al., 2021)	Rats (Adult)	Yes	Yes	Yes	ANOVA	Sex assessed statistically, appropriate controls included	
5	(Blanchard et al., 1987)	Rats (Juvenile)	Yes	Assessed, not significant	Yes	Multivariate ANOVA	Sex assessed statistically, appropriate controls included	
5	(Ieraci and Herrera, 2020)	Mice (Adult)	Yes	Yes	Yes	Multivariate ANOVA	Sex assessed statistically, appropriate controls included	
5	(Kenton et al., 2020)	Mice (Young Adult)	Yes	Yes	Yes	Multivariate ANOVA	Sex assessed statistically, appropriate controls included	
5	(Neese et al., 2004)	Rats (Young Adult)	Yes	Yes	Yes	Independent t-tests for each sex	Sex assessed statistically, appropriate controls included	
5	(Sickmann et al., 2014)	Rats (Adult)	Yes	Assessed, not significant	Yes	Multivariate ANOVA	Sex assessed statistically, appropriate controls included, ovariectomized females included in PAE analyses to determine estrogen influence	
5	(Subbanna and Basavarajappa, 2022)	Mice (Adult)	Yes	Assessed, not significant	Assessed, not significant	Multivariate ANOVA	Sex assessed statistically, appropriate controls included	
5	(Titterness and Christie, 2012)	Rats (Adolescent)	Yes	Yes	Yes	Multivariate ANOVA	Sex assessed statistically, appropriate controls included	
5	(Walter et al., 2022)	3xTg-AD Mice (Adult)	Yes	Yes	Yes	Multivariate ANOVA	Sex assessed statistically, appropriate controls included	
5	(Zimmerberg et al., 1991)	Rats (Adult)	Yes	Yes	Yes	Multivariate ANOVA	Sex assessed statistically, appropriate controls included	
4	(Patten et al., 2013)	Rats (Adult)	Yes	Yes	Yes	Multivariate ANOVA	Sexes assessed independently, appropriate controls included	
3	(Coles et al., 2011)	Humans (Young Adult)	Yes	Yes	Assessed in growth variables, not significant	Multivariate ANOVA, linear regression models, mediation analysis	Two PAE groups (with either Alcohol Related Neurodevelopmental Disorder [ARND] diagnosis or were dysmorphic) were statistically compared to an appropriate, non-PAE control	Sex by PAE interactions were not reported for cognitive outcomes. Sex included as a covariate for brain volume assessments. The PAE/ARND group exhibited slightly higher representation of females (72.2%) compared to the PAE/dysmorphic (53.3%) and control (53.8%) groups.
3	(Flannigan et al., 2023)	Humans (Adolescent and Adult)	Yes	Yes	No	Logistic regression, Pearson chi-square	Sex assessed statistically (in PAE subjects)	Study did not include control/non-PAE groups to facilitate assessment of PAE by sex interaction.
1	(Rangmar et al., 2015)	Humans (Adult)	Yes	Not assessed	Not assessed	Mann–Whitney *U* test	Sex ratio was matched between PAE and non-PAE controls	Sex was not included in statistical analyses, likely due to modest sample sizes among groups.

1 Studies were ranked on a scale of 1 to 5 based on strength of sex differences assessed. Points were assigned based on:

1) Were both sexes included? (Yes: 1pt; No/Unknown: 0 pts)

2) Were sexes directly compared to each other (statistical main effect of sex)? (Yes: 2 pts; No/Unknown: 0 pts)

2a) If not, were sexes assessed independently? (Yes: 1pt; No/Unknown: 0 pts)

3) Was a PAE by sex Interaction statistically assessed? (Yes: 2 pts; No/Unknown: 0 pts)

3a) If not, were PAE effects reported for each sex independently? (Yes: 1pt; No/Unknown: 0 pts)

**Table 3:** Referenced neurobehavioral studies investigating PAE and including both sexes, ranked and sorted by rigor in addressing sex as a biological variable.

Rigor Score^1^	Reference	Species (Age)	Includes Both Sexes	Main Effect of Sex	PAE by Sex Interaction	Statistics Performed	Strengths	Limitations
5	(Bake et al., 2021)	Rats (Adult)	Yes	Yes	Yes	ANOVA	Sex assessed statistically, appropriate controls included	
5	(Blanchard et al., 1987)	Rats (Juvenile)	Yes	Assessed, not significant	Yes	Multivariate ANOVA	Sex assessed statistically, appropriate controls included	
5	(Diaz et al., 2020)	Rats (Adolescent and Adult)	Yes	Yes	Yes	T-tests, ANOVA	Sex assessed statistically, appropriate controls included	
5	(Diaz et al., 2020)	Rats (Adult)	Yes	Established in prior publication (Diaz et al., 2016)	Yes	T-tests, ANOVA	Sex assessed statistically, appropriate controls included	
5	(Disney et al., 2008)	Humans (Adolescent)	Yes	Yes	Assessed, not significant	Multiple regression models	Sex assessed statistically, appropriate controls included	
5	(Hofmann et al., 2005)	Rats (Adult)	Yes	Yes	Yes	Multivariate ANOVA	Sex assessed statistically, appropriate controls included	
5	(Lynch et al., 2015)	Humans (Young Adult)	Yes	Yes	Yes	Sex included as independent variable in general linear models	Sex assessed statistically, appropriate controls included	
5	(Lynch et al., 2017)	Humans (Young Adult)	Yes	Yes	Yes	Sex included as independent variable in general linear models	Sex assessed statistically, appropriate controls included	
5	(Mooney and Varlinskaya, 2011)	Rats (Adolescent and Adult)	Yes	Assessed, not significant	Yes	Multivariate ANOVA	Sex assessed statistically, appropriate controls included	
5	(Neese et al., 2004)	Rats (Young Adult)	Yes	Yes	Yes	Independent t-tests for each sex	Sex assessed statistically, appropriate controls included	
5	(Panczakiewicz et al., 2016)	Humans (Child and Adolescent)	Yes	Yes	Assessed, not significant	Chi-square, Univariate and Multivariate ANOVA	Sex assessed statistically, appropriate controls included	
5	(Rouzer and Diaz, 2022)	Rats (Adolescent)	Yes	Yes	Yes	T-tests, ANOVA	Sex assessed statistically, appropriate controls included	
5	(Rouzer et al., 2017)	Rats (Adolescent)	Yes	Yes	Yes	Chi-square goodness of fit test, t-tests, ANOVA	Sexes assessed independently, appropriate controls included	
5	(Wang et al., 2023)	Rats (Adult)	Yes	Yes	Yes	Multivariate ANOVA, T-Tests	Sex assessed statistically, appropriate controls included	
3	(Streissguth et al., 2004)	Humans (Child to Adult)	Yes	Yes	No	Odds ratios, multivariate logistic regression	Sex assessed statistically, appropriate controls included	Odd ratios of risk factors for major outcomes were calculated, including sex as a risk factor; Two groups compared were individual with an FAS diagnosis or individuals wtih PAE but did not make criteria for an FAS diagnosis
3	(Coles et al., 2022)	Humans (Adult)	Yes	Yes	N/A	Path analysis (with sex as an exogenous variable ), chi-squared test, ANOVA	Paths from gender to mental health outcomes assessed statistically	Study included control/non-PAE groups; did not assess PAE by sex interaction; females make up greater percent of alcohol exposed (64.6%) group than alcohol affected (50.6%) and control (59.1%) groups
3	(Flannigan et al., 2023)	Humans (Adolescent and Adult)	Yes	Yes	No	Logistic regression, Pearson chi-square tests	Sex assessed statistically (in PAE subjects)	Study did not include control/non-PAE groups to fascilitate assessment of PAE by sex interaction
1	(Lees et al., 2020)	Humans (Preadolescent)	Yes	Unknown	Implied, but unknown	Sex included as a coviariate in mixed model & mediation analyses		Sex differences excluded from statistical analyses
1	(Olson et al., 1997)	Humans (Adolescent)	Yes	Unknown	Unknown	Sex included as an adjusted covariable in a regression model		Sex differences excluded from statistical analyses

1 Studies were ranked on a scale of 1 to 5 based on strength of sex differences assessed. Points were assigned based on:

1) Were both sexes included? (Yes: 1pt; No/Unknown: 0 pts)

2) Were sexes directly compared to each other (statistical main effect of sex)? (Yes: 2 pts; No/Unknown: 0 pts)

2a) If not, were sexes assessed independently? (Yes: 1pt; No/Unknown: 0 pts)

3) Was a PAE by sex Interaction statistically assessed? (Yes: 2 pts; No/Unknown: 0 pts)

3a) If not, were PAE effects reported for each sex independently? (Yes: 1pt; No/Unknown: 0 pts)

**Table 4:** Referenced alcohol seeking/preference studies investigating PAE and including both sexes, ranked and sorted by rigor in addressing sex as a biological variable.

Rigor Score^1^	Reference	Species (Age)	Includes Both Sexes	Main Effect of Sex	PAE by Sex Interaction	Statistics Performed	Strengths	Limitations
5	(Chotro and Arias, 2003)	Rats (Pre-weaning)	Yes	Assessed, not significant	Yes	ANOVA	Sex assessed statistically, appropriate controls included, multiple prenatal doses assessed	
5	(Diaz et al., 2020)	Rats (Adult)	Yes	Established in prior publication (Diaz et al., 2016)	Yes	T-tests, ANOVA	Sex assessed statistically, appropriate controls included	
5	(Griesler and Kandel, 1998)	Humans (Adolescent and Adult)	Yes	Implied, but not significant	Yes	Hierarchical multivariate logistic regression models.	Sex was reported as a non-significant predictor of adolescent drinking, prior to investigation of sex by PAE interactions	
5	(Kokhan et al., 2022)	Rats (Young Adult)	Yes	Yes	Yes	ANOVA	Sex assessed statistically, appropriate controls included	
5	(Lynch et al., 2017)	Humans (Young Adult)	Yes	Yes	Yes	Sex included as independent variable in general linear models	Sex assessed statistically, appropriate controls included	Females make up greater percentage of PAE groups than males
5	(Randall et al., 1983)	Mice (Adolescent)	Yes	Yes (*p* = 0.06)	Assessed, not significant	T-tests, ANOVA	Sex assessed statistically, appropriate controls included.	
3	(Goldschmidt et al., 2019)	Humans (Adult)	Yes	Implied	Unknown	Sex included as a coviariate in logistic regression models.	Sex (specifically, being male) was identified as a significant predictor in offspring drinking rates.	Sex by PAE interactions excluded from statistical analyses
3	(Gore-Langton and Spear, 2019)	Rats (Adolescent and Adult)	Yes	Unknown	Implied	ANOVA	Sexes assessed independently, appropriate controls included, multiple ethanol concentrations included in self-administration assessment.	Averages for ethanol consumption per group (sex/exposure) are reported in adults, but main effects of sex and interactions between sex by PAE are not stastistically reported; instead, effects of exposure are reported individually in each sex
3	(Pepino et al., 2004)	Rats (Adolescent)	Yes	Implied, but unknown	Implied	ANOVA	Sexes assessed independently, appropriate controls included	Sexes were assessed independently, but a main effect of sex was not reported. Effects of PAE within each sex differed, but a PAE by sex interaction was not reported
3	(Streissguth et al., 1996)	Humans (all ages)	Yes	Yes	No		Included sex-stratified estimate of alcohol/drug use prevalence	Report to CDC providing incidence rates of primary and secondary disabilities from individuals with PAE

1 Studies were ranked on a scale of 1 to 5 based on strength of sex differences assessed. Points were assigned based on:

1) Were both sexes included? (Yes: 1pt; No/Unknown: 0 pts)

2) Were sexes directly compared to each other (statistical main effect of sex)? (Yes: 2 pts; No/Unknown: 0 pts)

2a) If not, were sexes assessed independently? (Yes: 1pt; No/Unknown: 0 pts)

3) Was a PAE by sex Interaction statistically assessed? (Yes: 2 pts; No/Unknown: 0 pts)

3a) If not, were PAE effects reported for each sex independently? (Yes: 1pt; No/Unknown: 0 pts)

**Table 5:** Referenced brain imaging studies investigating PAE and including both sexes, ranked and sorted by rigor in addressing sex as a biological variable.

Rigor Score^1^	Reference	Species (Age)	Includes Both Sexes	Main Effect of sex	PAE by Sex Interaction	Statistics Performed	Strengths	Limitations
5	(Chen et al., 2012)	Humans (Late Adolescent/Young Adult)	Yes	Yes	Yes	Multivariate ANOVA	A control group (no PAE) was included in statistical analyses to determine differences from two distinct alcohol-exposed group	Females disproportionally represented (73%) in one PAE group
5	(Nardelli et al., 2011)	Humans (Child and Adolescent)	Yes	Yes	Yes	Univariate and Multivariate ANOVA	Sexes assessed independently, appropriate controls included	
4	(Uban et al., 2017)	Humans (Child and Adolescent)	Yes	Reported for hormone levels, but not overall imaging metrics	Yes	ANOVA & linear regression models	Sexes assessed independently, appropriate controls included	
1	(Archibald et al., 2001)	Humans (Adolescent)	Yes	Not assessed	Not assessed	Kruskal–Wallis and Mann–Whitney U tests	A control group (no PAE) was included in statistical analyses to determine differences from two alcohol-exposed groups: PAE and FAS	Sex was not included in statistical analyses, likely due to modest sample sizes among groups
1	(Astley et al., 2009)	Humans (Child to Adolescent)	Yes	Not assessed	Not assessed	ANOVA	A control group (no PAE) was included in statistical analyses to determine differences from three distinct alcohol-exposed groups	Sex was not included in statistical analyses, likely due to modest sample sizes among groups
1	(Cortese et al., 2006)	Humans (Preadolescent^2^ and adolescent)	Yes	Not assessed	Not assessed	Independent t-tests	A control group (no PAE) was included in statistical analyses to determine differences from alcohol-exposed subjects with or without a diagnosis of FAS	Sex was not included in statistical analyses, likely due to modest sample sizes among groups.
1	(Lees et al., 2020)	Humans (Preadolescent^2^)	Yes	Unknown	Implied, but unknown	Sex included as a coviariate in mixed model and mediation analyses		Sex differences excluded from statistical analyses
1	(Li et al., 2008)	Humans (Late Adolescent/Young Adult)	Yes	Not assessed	Not assessed	Group t-tests	A control group (no PAE) was included in statistical analyses to determine differences from alcohol-exposed subjects	Sex was not included in statistical analyses, likely due to modest sample sizes among groups
1	(Sowell et al., 2001)	Humans (Child and Adolescent)	Yes	Not assessed	Not assessed	T-tests	A control group (no PAE) was included in statistical analyses to determine differences from alcohol-exposed subjects	Sex was not included in statistical analyses, likely due to modest sample sizes among groups

1 Studies were ranked on a scale of 1 to 5 based on strength of sex differences assessed. Points were assigned based on:

1) Were both sexes included? (Yes: 1pt; No/Unknown: 0 pts)

2) Were sexes directly compared to each other (statistical main effect of sex)? (Yes: 2 pts; No/Unknown: 0 pts)

2a) If not, were sexes assessed independently? (Yes: 1pt; No/Unknown: 0 pts)

3) Was a PAE by sex Interaction statistically assessed? (Yes: 2 pts; No/Unknown: 0 pts)

3a) If not, were PAE effects reported for each sex independently? (Yes: 1pt; No/Unknown: 0 pts)

2 Preadolescent as the late childhood epoch directly preceding adolescence, including ages 9–11

**Table 6:** Referenced CVD and immune function studies investigating PAE and including both sexes, ranked and sorted by rigor in addressing sex as a biological variable.

Rigor Score^1^	Reference	Species (Age)	Includes Both Sexes	Main Effect of sex	PAE by Sex Interaction	Statistics Performed	Strengths	Limitations
5	(Bake et al., 2017)	Mice (Adult)	Yes	Assessed, not statistically significant	Assessed, not statistically significant	Univariate and Multivariate ANOVA	Sex assessed statistically, appropriate controls included, offspring assessed at multiple ages	
5	(Bake et al., 2021)	Rats (Adult)	Yes	Yes	Yes	ANOVA	Sex assessed statistically, appropriate controls included	
5	(Bake et al., 2022)	Rats (Adult into Middle Age)	Yes	Yes	Yes	ANOVA	Sex assessed statistically, appropriate controls included	
5	(Nguyen et al., 2014)	Rats (Adult)	Yes	Yes	Assessed, not statistically significant	ANOVA	Sex assessed statistically, appropriate controls included	
5	(Walton et al., 2019)	Rats (Middle Age Adult)	Yes	Yes	Yes	ANOVA	Sex assessed statistically, appropriate controls included	
5	(Weinberg and Jerrells, 1991)	Rats (Adult)	Yes	Yes	Yes	Multivariate ANOVA	Sex assessed statistically, appropriate controls included	
4	(Saha et al., 2021)	Rats (Adolescent and Adult)	Yes	Assessed, not statistically significant	Assessed, not statistically significant	ANOVA	Sexes assessed independently, appropriate controls included, offspring assessed during adolescence and adulthood	
3	(Weeks et al., 2020)	Humans and Zebrafish (Adult)	Yes	Unknown	Yes	ANOVA	Sexes assessed independently, appropriate controls included.	
1	(Ngai et al., 2008)	Sheep (Adult)	Yes	Not assessed	Not assessed	Logistic equation and F test.	Both sexes included in assessments, appropriate controls included	Sex was not included in statistical analyses, likely due to modest sample sizes among groups; males disproportionally represented (80%) in control group

1 Studies were ranked on a scale of 1 to 5 based on strength of sex differences assessed. Points were assigned based on:

1) Were both sexes included? (Yes: 1pt; No/Unknown: 0 pts)

2) Were sexes directly compared to each other (statistical main effect of sex)? (Yes: 2 pts; No/Unknown: 0 pts)

2a) If not, were sexes assessed independently? (Yes: 1pt; No/Unknown: 0 pts)

3) Was a PAE by sex Interaction statistically assessed? (Yes: 2 pts; No/Unknown: 0 pts)

3a) If not, were PAE effects reported for each sex independently? (Yes: 1pt; No/Unknown: 0 pts)

## Data Availability

No data was used for the research described in the article.
